# Dual targeting of CD19 and CD22 against B-ALL using a novel high-sensitivity aCD22 CAR

**DOI:** 10.1016/j.ymthe.2023.12.006

**Published:** 2023-12-15

**Authors:** Evangelia Kokalaki, Biao Ma, Mathieu Ferrari, Thomas Grothier, Warren Hazelton, Somayya Manzoor, Eren Costu, Julia Taylor, Anna Bulek, Saket Srivastava, Isaac Gannon, Ram Jha, Rosalind Gealy, Lukas Stanczuk, Tatiana Rizou, Mathew Robson, Mohamed El-Kholy, Vania Baldan, Matteo Righi, James Sillibourne, Simon Thomas, Shimobi Onuoha, Shaun Cordoba, Martin Pule

## Main text

(Molecular Therapy *31*, 2089–2104; July 2023)

In the originally published version of this article, the incorrect image was used in Figure 1A. The authors accidentally submitted the original version of the image, before the numbering was changed. However, the numbering of the domains has since been changed in the table of Figure 1B; thus the numbering of the domains in the Figure 1A image did not correlate with the table in Figure 1B. This inconsistency leads the reader to the wrong conclusion and is in conflict with the message from the text.

Figure 1 has been updated in the article online, and the authors apologize for any confusion this may have caused.

**Figure:**
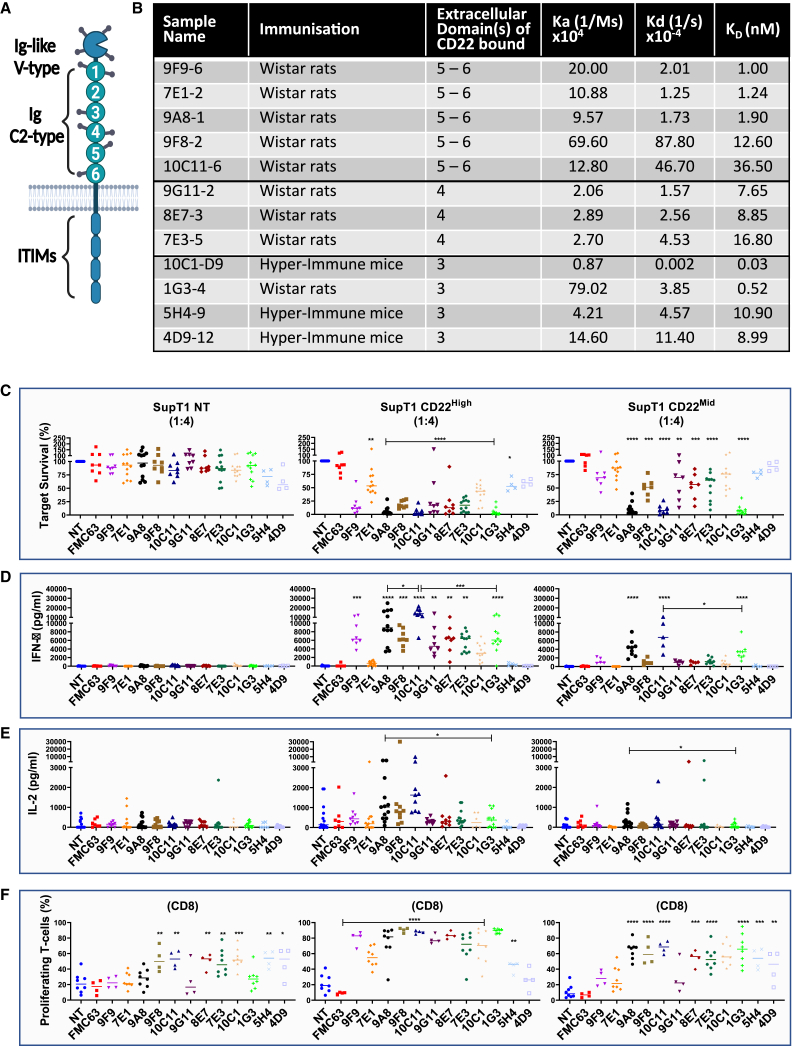
Figure 1. Screening of novel binders recognizing CD22 (corrected)

**Figure:**
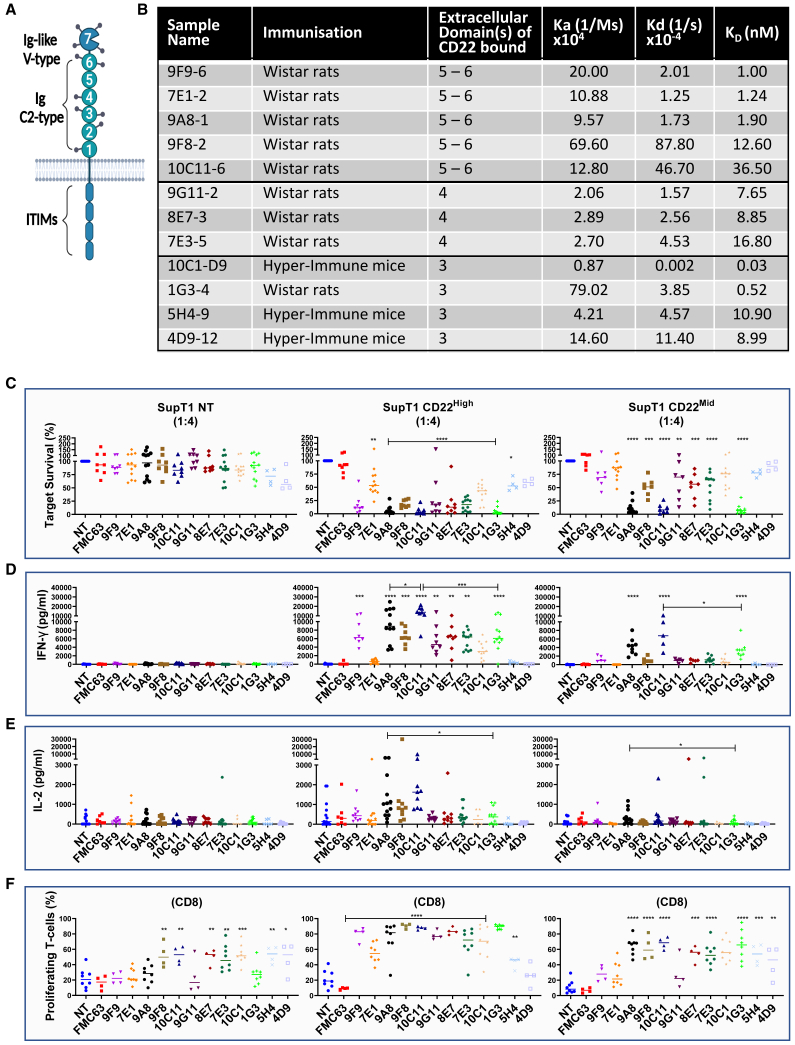
Figure 1. Screening of novel binders recognizing CD22 (original)

