# Supplementary material for: Dual targeting of CD19 and CD22 against B-ALL using a novel high-sensitivity aCD22 CAR
Source: Mol Ther. 2023 Mar 21;31(7):2089–104. doi: 10.1016/j.ymthe.2023.03.020 (PMC10362402; doi:10.1016/j.ymthe.2023.03.020)
Supplement: Document S1. Supplemental methods and Figures S1–S8 [file mmc1.pdf]

YMTHE, Volume *31*

Supplemental Information

Dual targeting of CD19 and CD22 against B-ALL using a novel high-sensitivity aCD22 CAR

Evangelia Kokalaki, Biao Ma, Mathieu Ferrari, Thomas Grothier, Warren Hazelton, Somayya Manzoor, Eren Costu, Julia Taylor, Anna Bulek, Saket Srivastava, Isaac Gannon, Ram Jha, Rosalind Gealy, Lukas Stanczuk, Tatiana Rizou, Mathew Robson, Mohamed El-Kholy, Vania Baldan, Matteo Righi, James Sillibourne, Simon Thomas, Shimobi Onuoha, Shaun Cordoba, and Martin Pule

Supplemental Methods

Surface plasmon resonance

Recombinant anti-CD22 antibodies in IgG format, were captured on flow cells 2, 3 and 4 on a Series S Protein A sensor chip (Cytiva 29650263) to a density of 40-60 RU using a Biacore T200 instrument. HBS-P+ buffer was used as running buffer is all experimental conditions. Recombinant purified CD22 (Acro Biosystems SI2-H5228) at known concentrations was used as the 'analyte' and injected over the respective flow cells with 150 s contact time and 300s dissociation at 30 µl/minute of flow rate with a constant temperature of 25°C. In each experiment, flow cell 1 was unmodified and used for reference subtraction. A '0 concentration' sensogram of buffer alone was used as a double reference subtraction to factor for drift. Data were fit to a 1:1 Langmuir binding model. Since a capture system was used, a local Rmax parameter was used for the data fitting in each case.

Epitope binning

Recombinant anti-CD22 antibodies were immobilized on flow cell 2 of channels 1, 3, 5 and 7 of a Series S CM5 sensor chip (Cytiva 29104988) to a density of 820-1350 RU using a Biacore 8k instrument. HBS-P+ buffer was used as running buffer is all experimental conditions. Recombinant purified CD22 (Acro Biosystems SI2-H5228) at 200 nM (analyte 1) was injected over the flow channels for 150s at 30 µl/min, followed by 100 nM of the challenging anti-CD22 antibody (analyte 2) for 150s at 30 µl/min. A dissociation phase of 300s was included at the end. A condition without analyte 2 injection was used as baseline to assess analyte 2 contribution to RU. A '0 concentration' sensogram of buffer alone was used as reference subtraction to factor for drift.

Cell line engineering with STOP/SKIP

In order to obtain low CD22 expressing cells, we introduced a STOP/SKIP sequence upstream of the transgene. A STOPSKIP motif is a DNA sequence that bears a stop codon followed by a read-through sequence (e.g. CATG), with the aim of reducing the translational efficiency of any downstream transgene (Loughran et al. 2014). Different STOP codons led to different expression stringency and thus expression levels.

Antigen density measurement

Antigen density was determined by labelling the cell lines with aCD22 (BioLegend; 302506), aCD19 (BioLegend; 302208), or isototype (BioLegend; 400112) antibodies conjugated to PE. Concomitantly, we ran Quantibrite beads (BD Biosciences; 10626384), based on the beads' fluorescence and know density we created a standard curve. For each sample, the median fluorescence for PE was measured and their density calculated based on the Quantibrite standard curve.

Exhaustion phenotyping

Exhaustion phenotyping was performed by labelling transduced T cells for Tim3 (BioLegend; 345008), Lag-3 (Enzo; ALX-804-806F-C100), and PD-1 (BioLegned; 621608) exhaustion markers. $0.5x10^5$ T cells were cultured at a starvation assay in the absence of antigenic stimulus or IL-2 for 6 days. The exhaustion severity was measured by the amount of markers expressed on the T cells (single, double, triple).

Supplemental Methods

Restimulation Assay

We set-up a co-culture of $5x10^4$ effectors in the presence of $5x10^4$ targets (SupT1 NT, SupT1 $CD22^{Mid}$, or $CD22^{Low}$). Fresh targets were introduced every 4-6 days for stimulation 2 and 3. Stimulation 1 (Stim 1) was measured on day 3 after the assay set-up. The lysis has been normalised to that of SupT1 NT for each time-point.

Engineering Nalm6 $CD19^{KO}$

Nalm-6 cells (DSMZ clone ACC 128) were transduced with retrovirus encoding blasticidin S deaminase and Streptococcus pyogenes Cas9. Selection for transduced cells was carried out by culturing the cells in 20 mg/mL blasticidin (Invivogen) for 2 weeks. To generate a CD22 knockout, 1x106 Cas9-expressing Nalm-6 cells were nucleofected, using SF buffer and pulse code CV104 (Lonza), with 100 pmol of sgRNA (IDT) targeting the CD22 gene (5'-GAAACCCTCTACGCCTGGGA-3'). Single cell clones were established and then transduced with retrovirus encoding the sort select marker RQR8 and chimeric CD22/CD19, consisting of the CD22 ectodomain fused to the CD19 transmembrane and truncated cytoplasmic domain (residues 333-556 deleted). These cells were nucleofected with sgRNA (IDT) targeting the CD19 gene (5'-TGGAATGTTTCGGACCTAGG-3') to generate CD19 knockout cells.

9A8 Sequence

aCD22_9A8_VK:
DIQMTQSPSSLSASLGDRVTITCRSSQDIGNYLTWFQQKVGRSPRRMIYGAIKLEDGVPSRFSGSR
SGSDYSLTISSLESEDVADYQCLQSIQYPFTFGSGTKLEIKR
aCD22_9A8_VH:
EVQLVESGGGLVQPGRSLKLSCAASGFTFSNFAMAWVRQPPTKGLEWVASISTGGGNTYYRDSV
KGRFTISRDDAKNTQYLQMDSLRSEDTATYYCARQRNYYDGSYDYEGYTMDAWGQGTSVTVS

References

Loughran, G., Chou, M.-Y., Ivanov, I.P., Jungreis, I., Kellis, M., Kiran, A.M., Baranov, P.V., and Atkins, J.F. (2014). Evidence of efficient stop codon readthrough in four mammalian genes. Nucleic Acids Res. *42*, 8928–8938.

Figure S1: Novel aCD22 binders and their biophysical properties

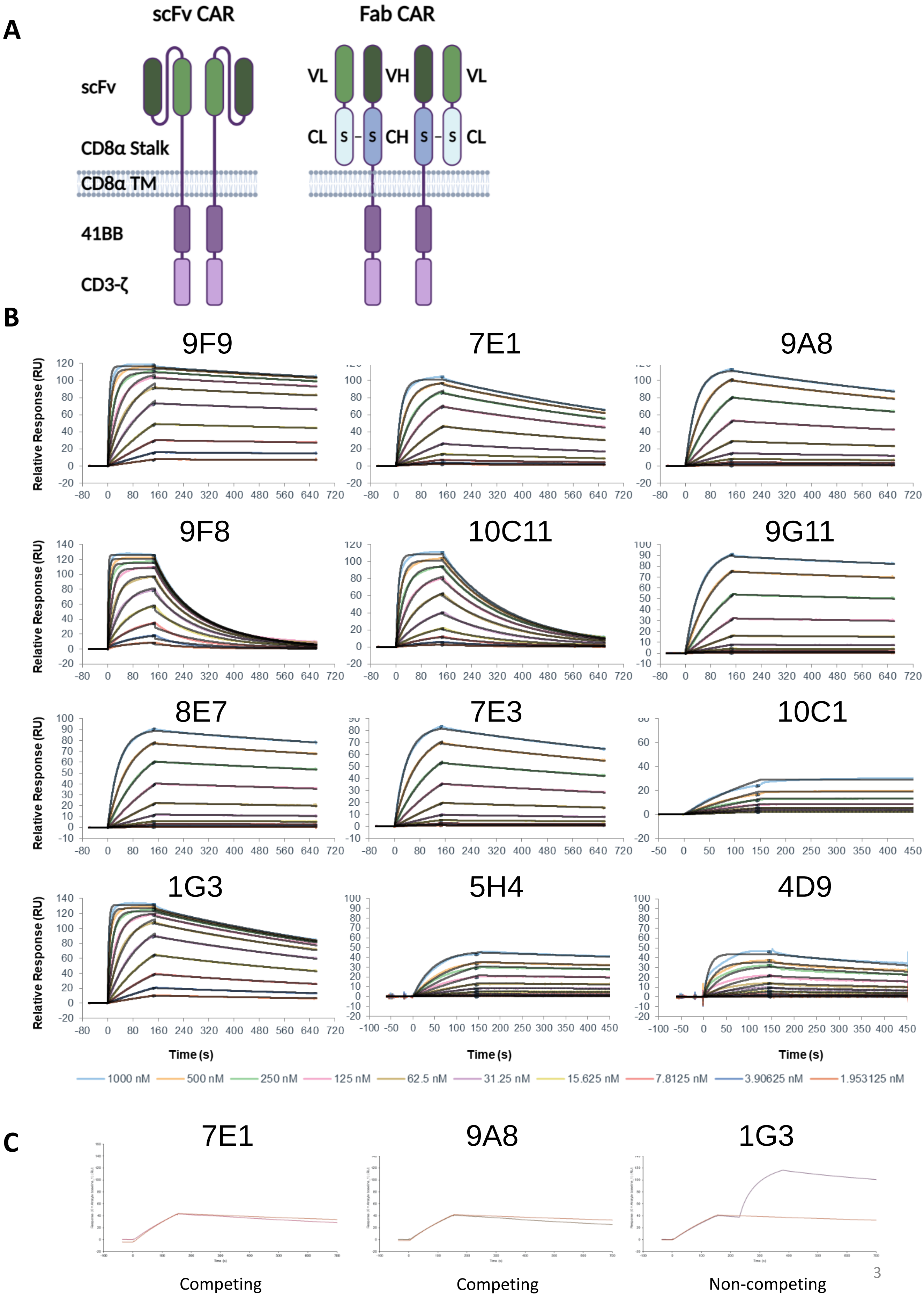

Figure S1: Novel aCD22 binders and their biophysical properties

A) The novel aCD22 CARs were screened into a Fab format, wherein the heavy and light chains were expressed separately. The light chain was fused to the IgK constant kappa domain, while the heavy chain was fused to the constant heavy chain of IgG followed by CD28 transmembrane, 4-1BB and CD3ζ. **B)** The kinetics of the binders were determined with Biacore technology. **C)** Epitope mapping was performed utilising 9A8.

Figure S2: Engineered SupT1 cells to express high and low densities of CD22

A

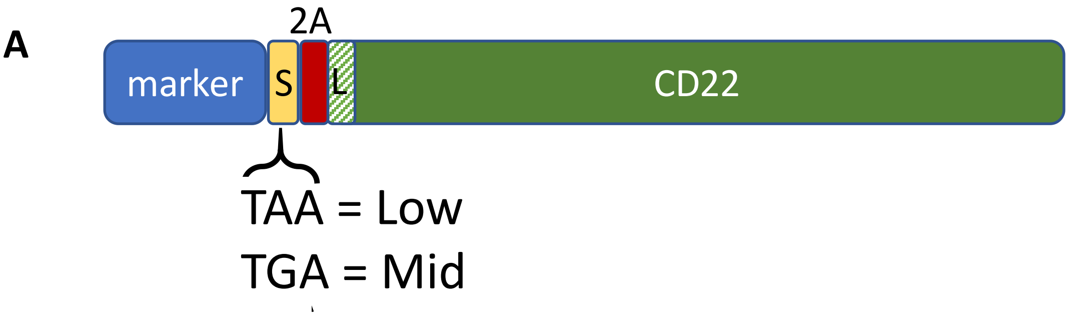

B

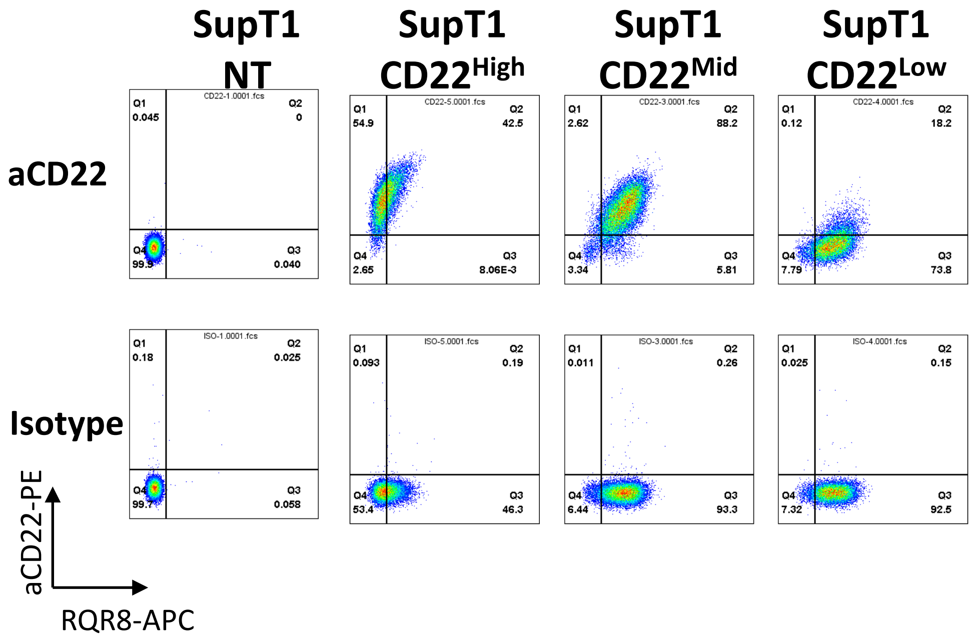

C

Cell Line	Density (Molecules / cell)
SupT1 CD22 High	6,603
SupT1 CD22 Mid	3,594
SupT1 CD22 Low	490

E

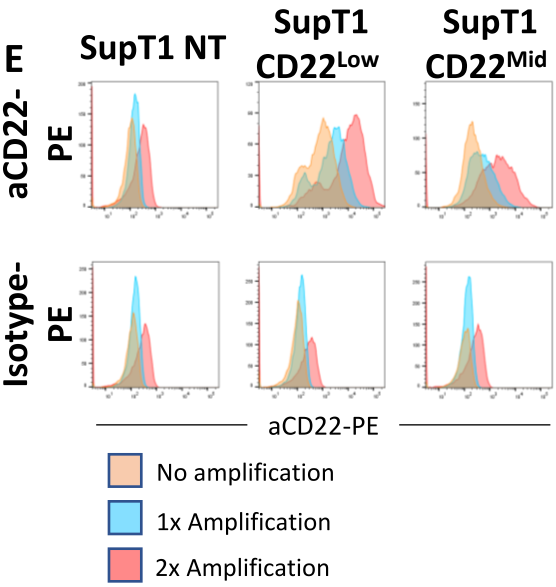

D

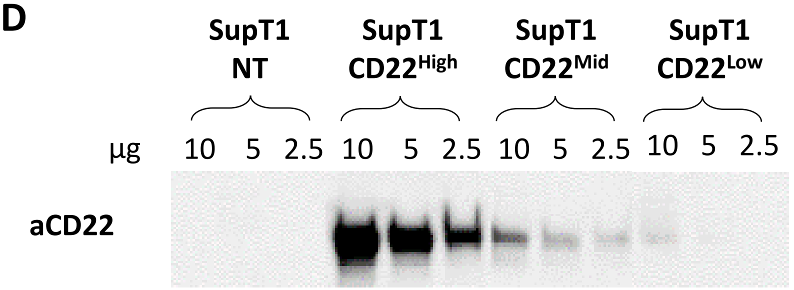

Figure S2: Engineered SupT1 cells to express high and low densities of CD22

A) In order to obtain low expressing CD22 target cells lines we introduced the STOP/SKIP technology, which comprises a STOP codon followed by a SKIP sequence leading to the ribosome skipping the STOP codon. The STOP/SKIP upstream of the transgene reduces the expression of the transgene. **B)** The expression of CD22 on the engineered SupT1 was measured by flow cytometry. The cells were labelled with either isotype control or aCD22 antibody, as well as the marker gene upstream of the transgene was labelled to ascertain the cassette expression. **C)** The antigen density was calculated based on the flow cytometry while concomitantly using with Quantibrite beads to obtain a standard curve. The CD22 level on SupT1 CD22^{Low} was only subtly detectable by flow cytometry. **D)** Hence, we run western blot with different concentration of cell lysate loaded to investigate the presence of CD22 in SupT1 CD22^{Low}. **E)** Finally, in order to ascertain the presence of CD22 on the SupT1 by flow cytometry, we amplified the signal using QIFIKIT kit for one or two rounds of amplification.

Figure S3: Binder Screening

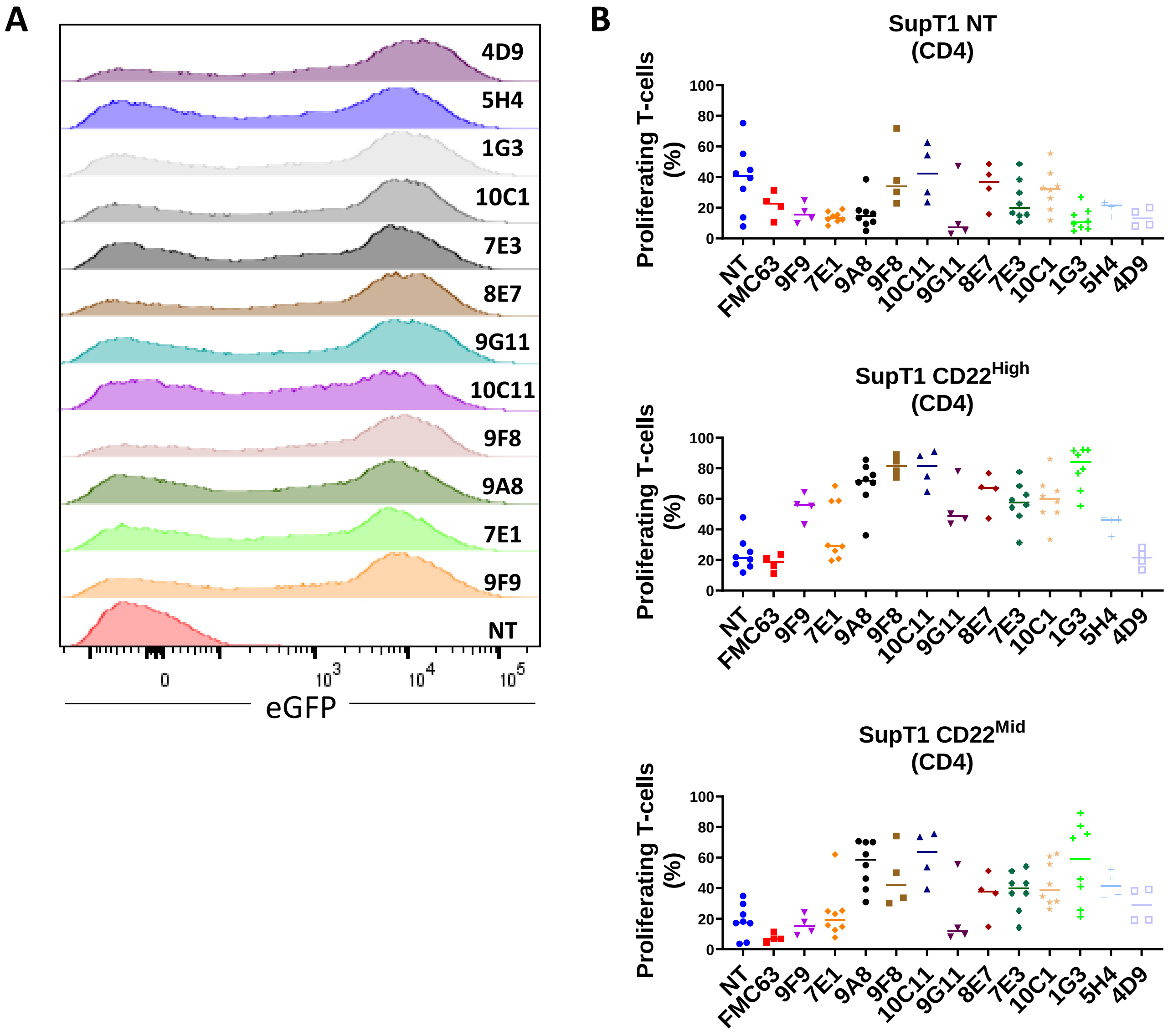

Figure S3: Binder Screening

A) The expression of the aCD22 binders was validated by the expression of the cassette marker gene, eGFP. **B)** T cells were labelled with CellTrace Violet and cultured in the presence of non-transduced SupT1 cells, or SupT1 engineered to express high and low levels of CD22 at a ratio of 1:1 for 4 days. The populations shown is the transduced proliferating CD4 compartment.

Figure S4: Comparison of 9A8 to M971 in lentiviral platforms

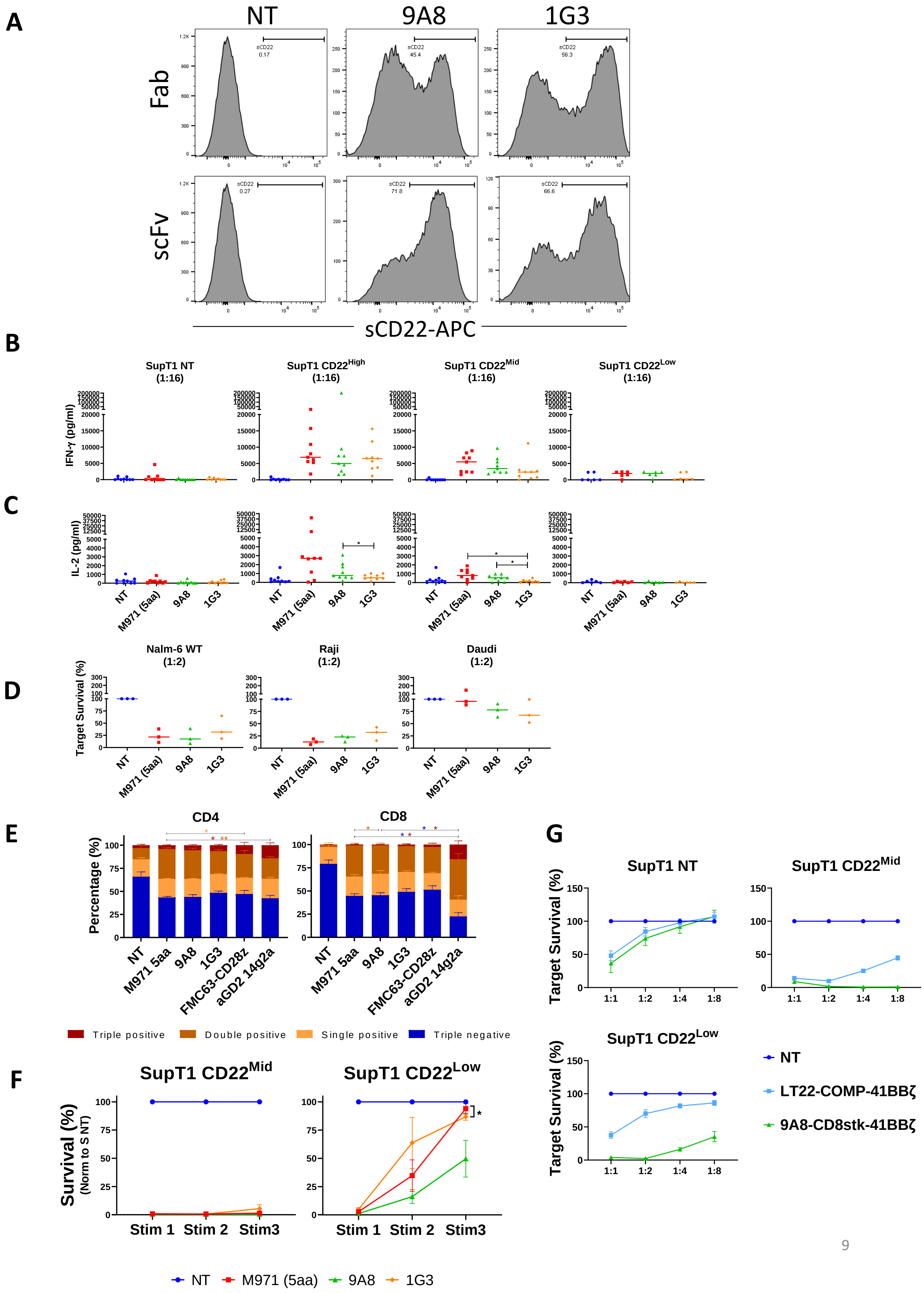

Figure S4: Comparison of 9A8 to M971 in lentiviral platforms

A) The expression of either scFv or Fab bearing CARs was assessed by the labelling with soluble CD22 antigen. The cytokine production of the M971, 9A8 and 1G3 CARs in the scFv lentiviral format was measured by ELISA for the ratio 1:16. The cytokines measured were IFN-γ **(B)** or IL-2 **(C)**. **D)** The CAR cytotoxicity was measured against B-cell lines, such as Nalm-6, Raji, and Daudi cells at an E:T of 1:2 at 72h. **E)** We assessed the exhaustion profile of the CARs by labelling for the exhaustion markers: PD-1, Tim3 and Lag3, and measuring the severity of exhaustion by the number of markers concomitantly expressed. The exhaustion read-out was flow-cytometry based on day 6 after a starvation assay. **F)** The CAR T cells were challenged in a re-stimulation assay, wherein the T cells were stimulated with SupT1 $CD22^{Mid}$ or $CD22^{Low}$ target cells three consecutive times. **G)** 9A8 was compared to LT22 at E:T ratios of 1:1, 1:2, 1:4, and 1:8. All statistical analyses were carried out with Student's t-tests. (n=3-9)

Figure S5: *In vivo* comparison of 9A8 to M971 in lentiviral platforms

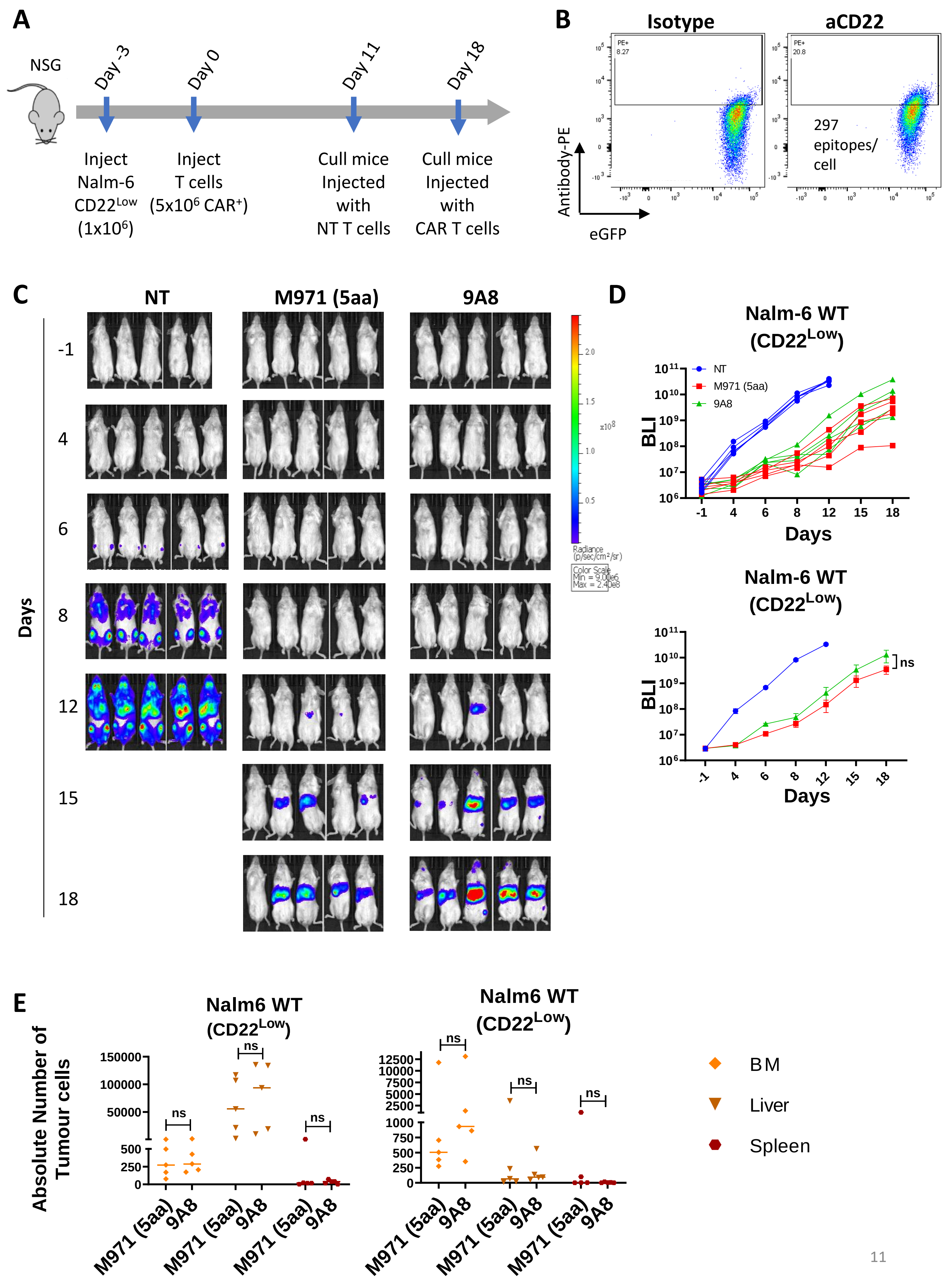

Figure S5: *In vivo* comparison of 9A8 to M971 in lentiviral platforms

A) Schematic representation of the *in vivo* NSG model. **B)** The CD22 antigen profile of Nalm-6 wt ($CD22^{Low}$) was validated by isotype or aCD22-PE labelling and measured with Quantibrite beads. BLI was performed to measure the tumour burden up to day 18 after T cells injection as shown in **(C)**. **D)** The BLI measurements were plotted to compare the anti-tumour efficacy between M971 and 9A8. **E)** The tumour cells and CAR T cells were enumerated by flow cytometry on day 18 for bone marrow, liver, and spleen tissues. All statistical analyses were carried out with Student's t-tests. (n=5 mice, T cells derived from 1 donor)

Figure S6: Co-transduction of CAT and 9A8

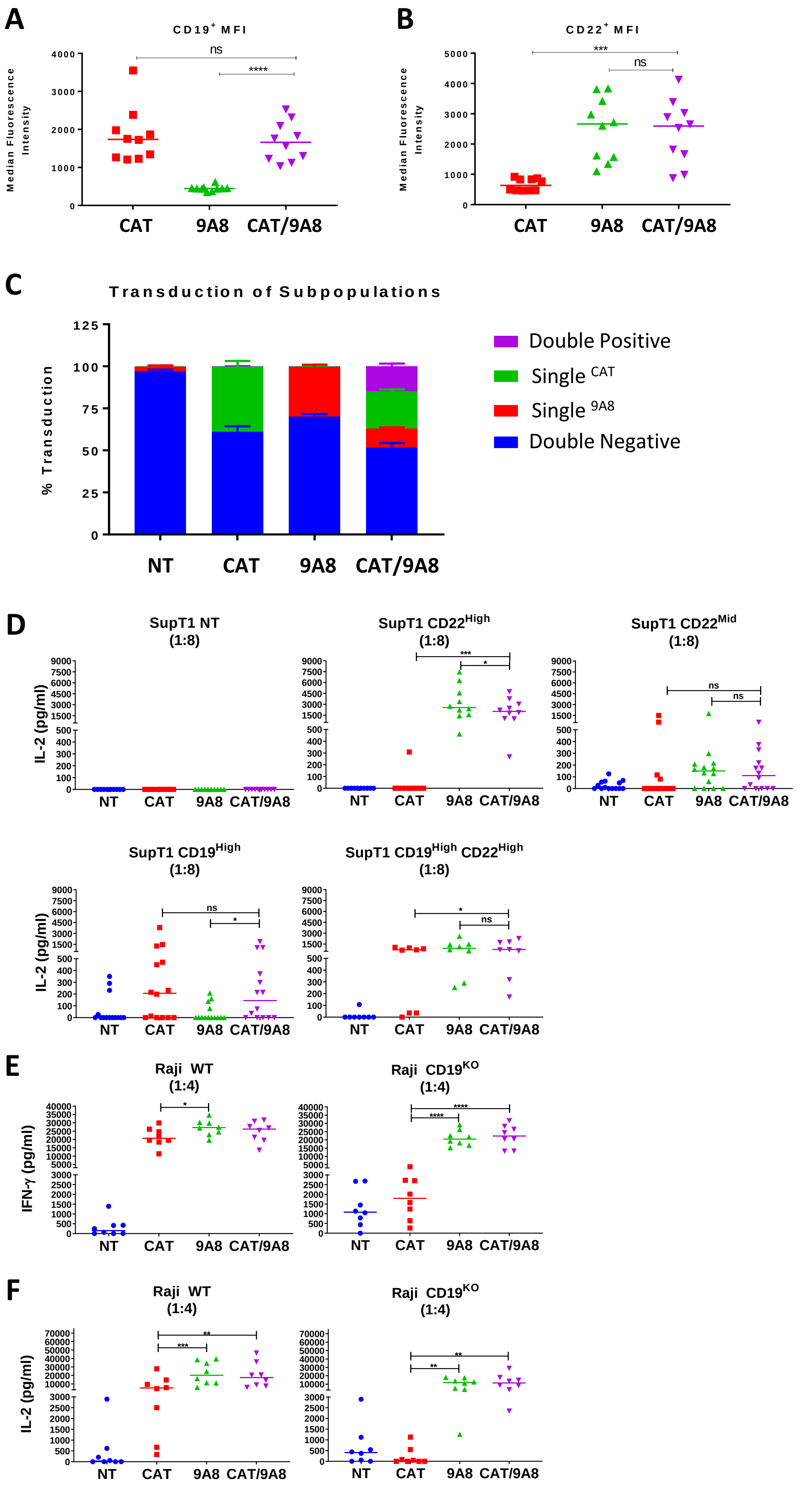

Figure S6: Co-transduction of CAT and 9A8

The MFI for the expression of the CAT **(A)** or 9A8 **(B)** CAR in the single or double transduction conditions was assessed by labelling the cells with anti-CAT idiotype and soluble CD22. The expression level of either CAR in the CAT/9A8 product was comparable to the single CAR conditions. The prevalence of each CAR subpopulation expressed in CAT/9A8 is shown in **(C)**, wherein Single^{CAT} or Single^{9A8} or double expressing subpopulations are shown in red, green or purple, respectively. **D)** The IL-2 release capacity was measured in response to CD22 mid or high expressing SupT1 targets at 1:8 E:T at a 72h time-point. Both IFN-γ **(E)** and IL-2 **(F)** secretion of the CARs against Raji WT and Raji CD19^{KO} (1:2 E:T) cells was quantified by ELISA at 72h. All statistical analyses were carried out with Student's t-tests. (n=8)

Figure S7: Engineering Nalm-6 in order to knock-out CD19

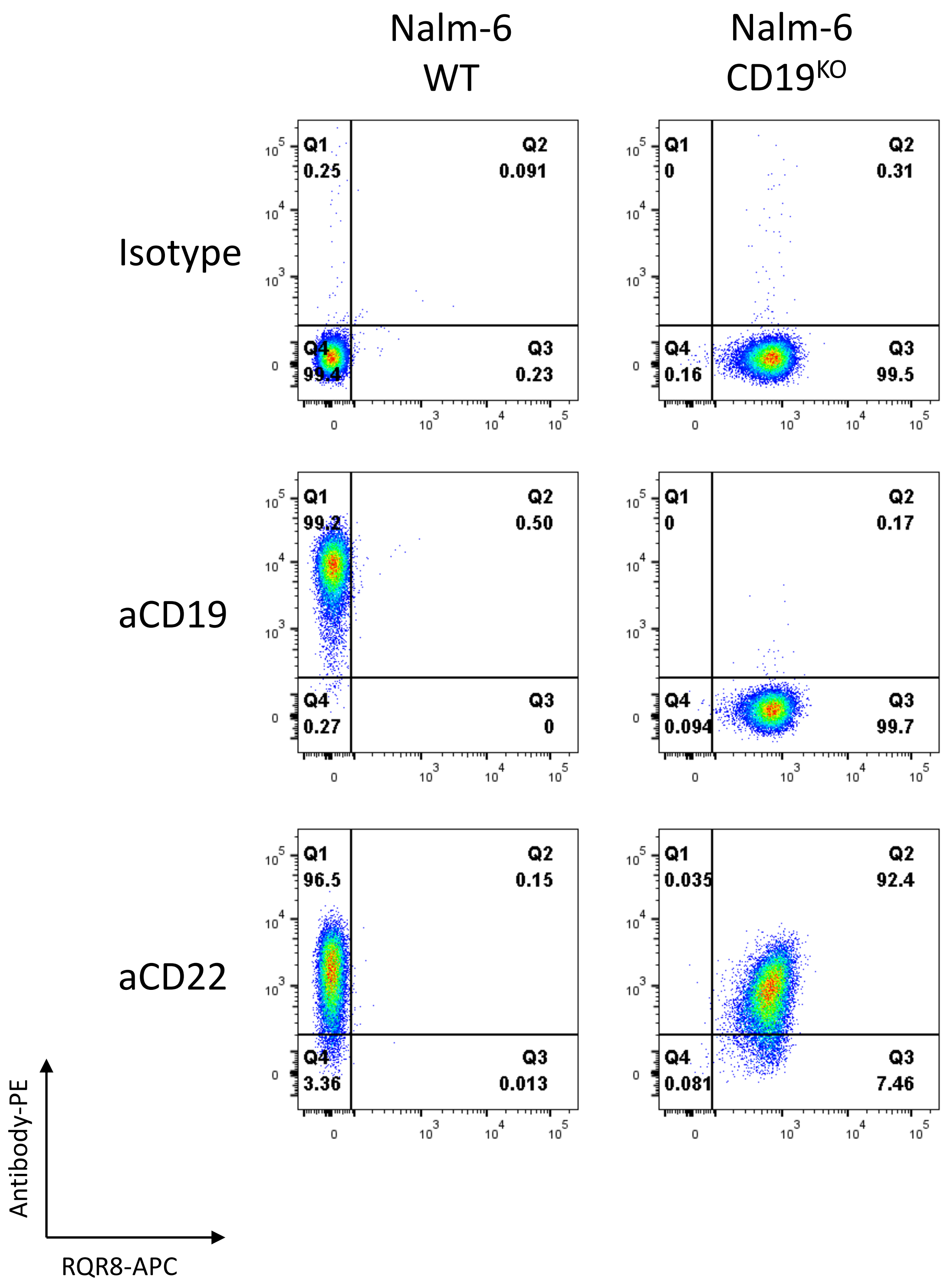

Figure S7: Engineering Nalm-6 in order to knock-out CD19

CD19 was downregulated in Nalm-6 WT cells by CRISPR technology. Flow-cytometry assessment demonstrated the lack of CD19 expression in the engineered Nalm-6 $CD19^{KO}$ cells, while the expression of CD22 antigen is still present.

Figure S8: *In vivo* data for CAT/9A8

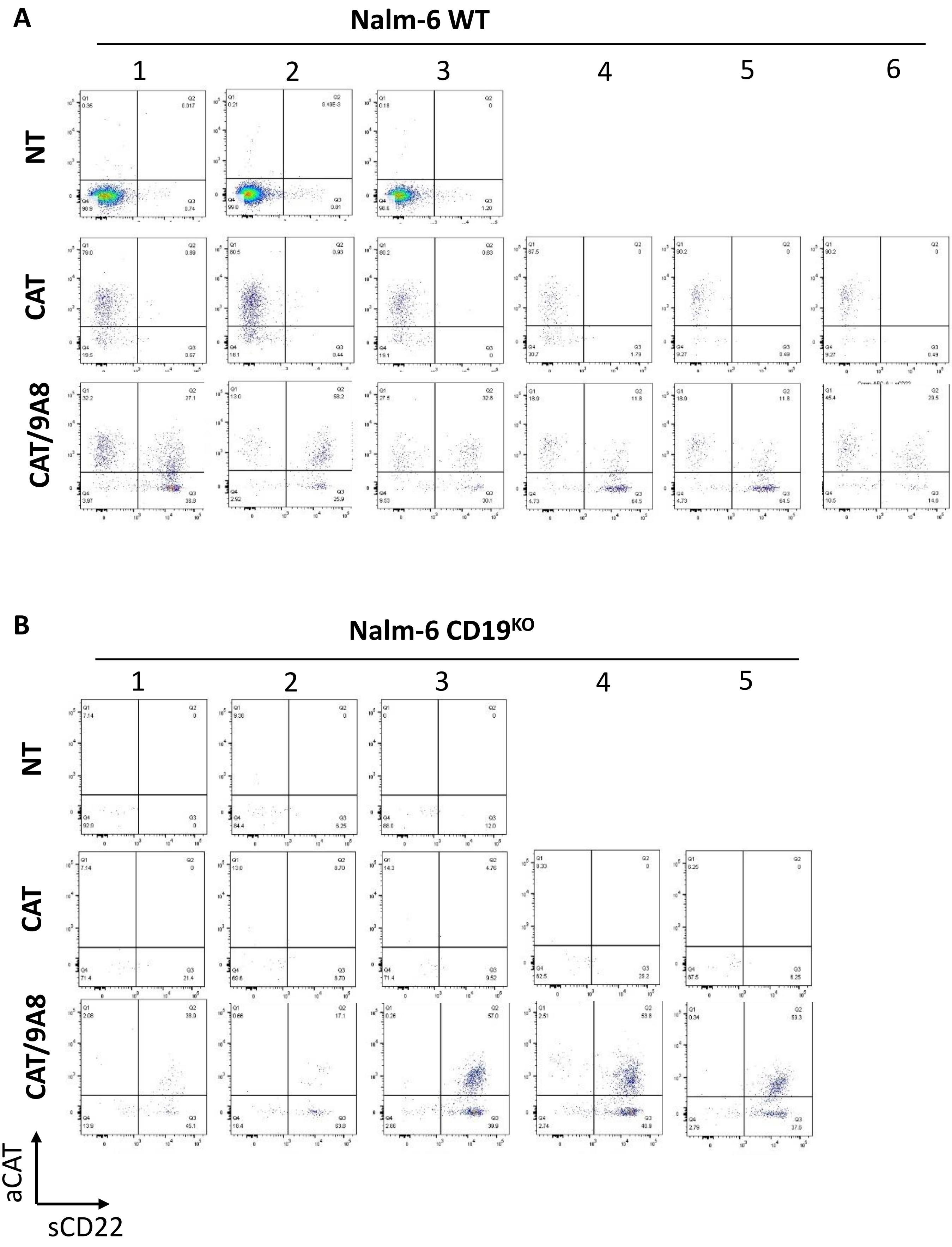

Figure S8: *In vivo* data for CAT/9A8

A) NSG mice were injected with 10^6 Nalm-6 WT cells at day -4 and at day 0 were inoculated with $5x10^6$ transduced CAR T cells. **B)** Nalm-6 $CD19^{KO}$ were utilised to simulate an antigen escape model. 10^6 tumour cells were injected 8 days prior to the T cell inoculation of $5x10^6$ transduced CAR T cells. Bone marrow from mice culled at day 14 were homogenised and labelled for the CAR bearing human T cells. The CAR expression was validated by labelling with anti-CAT idiotype and soluble CD22 for both Nalm-6 WT **(A)** and $CD19^{KO}$ **(B)** cells.
