# Supplementary material for: Dual targeting of CD19 and CD22 against B-ALL using a novel high-sensitivity aCD22 CAR
Source: Mol Ther. 2023 Mar 21;31(7):2089–104. doi: 10.1016/j.ymthe.2023.03.020 (PMC10362402; doi:10.1016/j.ymthe.2023.03.020)
Supplement: Document S2. Article plus supplemental information [file mmc2.pdf]

Molecular Therapy
Original Article

Dual targeting of CD19 and CD22 against B-ALL using a novel high-sensitivity aCD22 CAR

Evangelia Kokalaki,[1] Biao Ma,[1] Mathieu Ferrari,[1] Thomas Grothier,[1] Warren Hazelton,[1] Somayya Manzoor,[1] Eren Costu,[1] Julia Taylor,[1] Anna Bulek,[1] Saket Srivastava,[1] Isaac Gannon,[1] Ram Jha,[1] Rosalind Gealy,[1] Lukas Stanczuk,[1] Tatiana Rizou,[1] Mathew Robson,[1] Mohamed El-Kholy,[1] Vania Baldan,[1] Matteo Righi,[1] James Sillibourne,[1] Simon Thomas,[1] Shimobi Onuoha,[1] Shaun Cordoba,[1] and Martin Pule[1,2]

[1]Autolus Ltd, The MediaWorks, 191 Wood Ln, London W12 7FP, UK; [2]Department of Haematology, University College London, 72 Huntley Street, London WC1E 6BT, UK

CAR T cells recognizing CD19 effectively treat relapsed and refractory B-ALL and DLBCL. However, CD19 loss is a frequent cause of relapse. Simultaneously targeting a second antigen, CD22, may decrease antigen escape, but is challenging: its density is approximately 10-fold less than CD19, and its large structure may hamper immune synapse formation. The characteristics of the optimal CD22 CAR are underexplored. We generated 12 distinct CD22 antibodies and tested CARs derived from them to identify a CAR based on the novel 9A8 antibody, which was sensitive to low CD22 density and lacked tonic signaling. We found no correlation between affinity or membrane proximity of recognition epitope within Ig domains 3–6 of CD22 with CART function. The optimal strategy for CD19/CD22 CART co-targeting is undetermined. Co-administration of CD19 and CD22 CARs is costly; single CARs targeting CD19 and CD22 are challenging to construct. The co-expression of two CARs has previously been achieved using bicistronic vectors. Here, we generated a dual CART product by co-transduction with 9A8-41BBζ and CAT-41BBζ (obe-cel), the previously described CD19 CAR. CAT/9A8 CART eliminated single- and double-positive target cells *in vitro* and eliminated CD19⁻ tumors *in vivo*. CAT/9A8 CART is being tested in a phase I clinical study (NCT02443831).

INTRODUCTION

CD19-directed chimeric antigen receptor (CAR) T cell therapy has shown remarkable success in the treatment of refractory B cell malignancies.[1–3] The emergence of CD19 negative tumor escape is a frequent cause of relapse with a reported incidence between 25% and 70%.[2,4–6] One strategy to prevent CD19-negative tumor escape is the simultaneous targeting of CD19 and a second B lineage antigen.

CD22 is expressed early in B cell ontogeny and continues to be expressed until differentiation into plasma cells.[7,8] CD22 is expressed by B cell acute lymphoblastic leukemia (B-ALL),[9–12] with 50%–100% of adult B-ALL[13–15] and 90% pediatric B-ALL malignant cells being positive.[16,17] CD22 is also expressed in other B cell malignancies such as mantle cell lymphoma,[18] follicular lymphoma, and diffuse large B cell lymphoma (DLBCL).[19] Further, clinical experience demonstrates efficacy of CD22 CAR in B-ALL and DLBCL.[20,21]

However, CD22 CAR design faces two challenges: first, CD22 density is modest, with a median density of 2,839–3,470 molecules per cell,[12] which is further decreased after CAR targeting.[20] Second, CD22 has a large, rigid, heavily glycosylated ectodomain[22] comprising seven immunoglobular domains.[23] The size and rigidity of the ectodomain can compromise immune synapse formation and hence impede CAR function.[24]

Binder requirements for optimal CD22 CAR function are not well established. Most pre-clinical and clinical exploration of CD22 CAR function has been performed using CARs derived from the human phage library antibody M971.[25] To increase this experience, we developed a library of novel CD22 CARs, each based on a unique binder, of which 12 were interrogated for *in vitro* efficacy and sensitivity, allowing for the selection of an optimal CD22 CAR.

The optimal strategy for CAR targeting of two antigens has not been established. Clinical studies using a two-CAR approach against CD19 and CD22 have been successful, but require the manufacture of two products.[26] Alternative approaches using single CARs that express two scFv are technically challenging, with recent disappointing clinical data.[27]

We have previously described CAT-41BBζ (obe-cel), a CD19 CAR designed to improve engraftment and decrease toxicity.[28,29] We next explored a co-transduction approach to co-express CAT-41BBζ with the newly developed CD22 CAR. This approach allowed the generation of a CD19/CD22-specific CAR T product with activity against both antigens *in vitro* and *in vivo*.

Received 3 September 2022; accepted 16 March 2023;
https://doi.org/10.1016/j.ymthe.2023.03.020.

Correspondence: Martin Pule, Department of Haematology, University College London, 72 Huntley Street, London WC1E 6BT, UK.
E-mail: m.pule@ucl.ac.uk

This is an open access article under the CC BY license (http://creativecommons.org/licenses/by/4.0/).

A

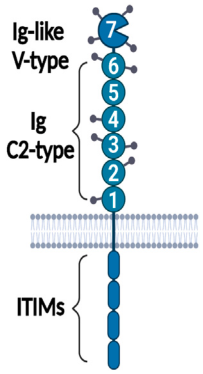

B

Sample Name	Immunisation	Extracellular Domain(s) of CD22 bound	Ka (1/Ms) x10[4]	Kd (1/s) x10[-4]	K_D (nM)
9F9-6	Wistar rats	5 – 6	20.00	2.01	1.00
7E1-2	Wistar rats	5 – 6	10.88	1.25	1.24
9A8-1	Wistar rats	5 – 6	9.57	1.73	1.90
9F8-2	Wistar rats	5 – 6	69.60	87.80	12.60
10C11-6	Wistar rats	5 – 6	12.80	46.70	36.50
9G11-2	Wistar rats	4	2.06	1.57	7.65
8E7-3	Wistar rats	4	2.89	2.56	8.85
7E3-5	Wistar rats	4	2.70	4.53	16.80
10C1-D9	Hyper-Immune mice	3	0.87	0.002	0.03
1G3-4	Wistar rats	3	79.02	3.85	0.52
5H4-9	Hyper-Immune mice	3	4.21	4.57	10.90
4D9-12	Hyper-Immune mice	3	14.60	11.40	8.99

C

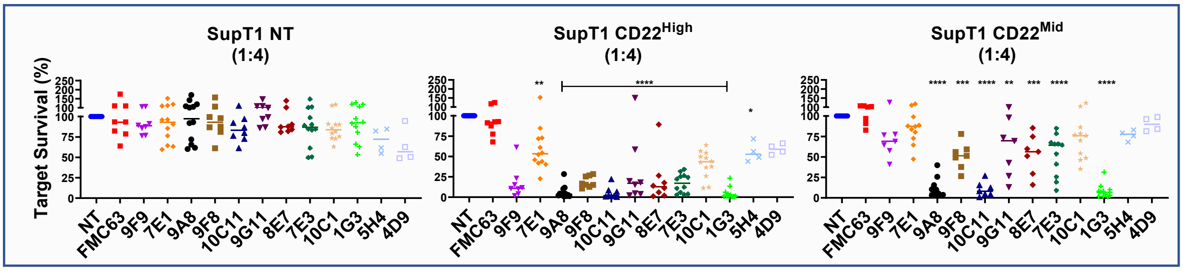

D

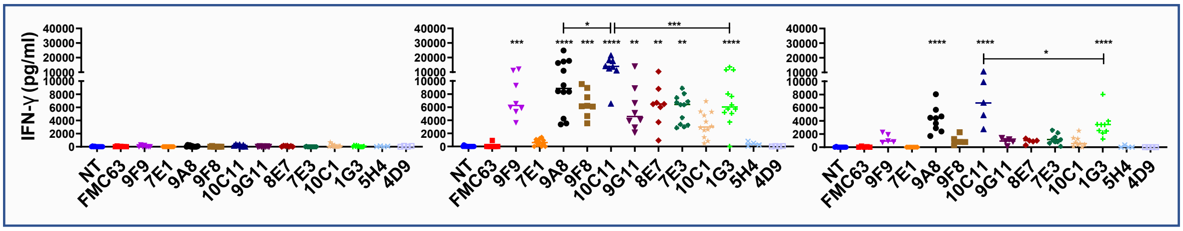

E

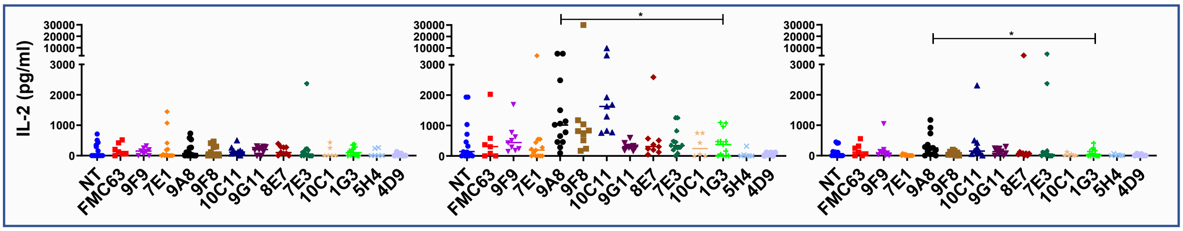

F

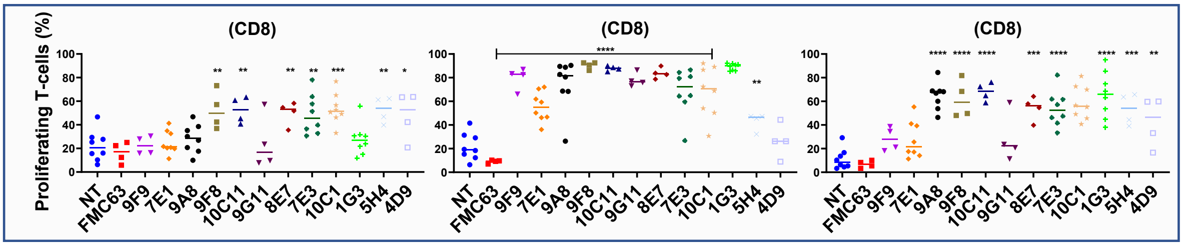

(legend on next page)

RESULTS

Screening novel CD22-targeting CARs for efficacy against low-density targets

CD22 has a tall, bulky ectodomain (Figure 1A). We discovered a set of novel CD22-specific antibodies, which were derived from hybridomas from immunized Abveris DiversimAb mice and Wistar rats.[30,31] Only antibodies binding the proximal 3–6 Ig domains of CD22 were characterized further. Five antibodies bound domains 5–6, three bound domain 4, and four bound domain 3. The affinity ranged between 28 pM and 36.5 nM (Figure S1B). The biophysical properties and identification of the domain recognized are summarized in Figure 1B. These antibodies were next formatted into CARs. To avoid the requirement for optimization of scFv orientation and linker lengths, a Fab 41BB-ζ CAR format was chosen for screening (Figure S1A).[32]

Functional screening was performed using primary human T cells transduced to express the CD22 CARs. Our interest was focused on identifying CARs that are functional against low antigen density and high disease burden. Hence, we engineered SupT1 cells to express the pathological density of CD22 (3,594 copies per cell) as target cells (SupT1 $CD22^{Mid}$). SupT1 target cells engineered to express CD22 (6,603 molecules/cell) were also used as targets (SupT1 $CD22^{High}$) (Figure S2).

The level of expression for the 12 CD22 CARs was comparable as determined by flow cytometric analysis through labeling with soluble CD22 (sCD22). The majority of the CD22 CARs triggered lysis of SupT1 $CD22^{High}$ targets (Figure 1C), however only three (9A8, 10C11, and 1G3) eliminated SupT1 $CD22^{Mid}$. The same three CARs triggered IFN-γ release against SupT1 $CD22^{Mid}$ (Figure 1D), whereas only 9A8 produced modest levels of IL-2 against the mid-density target (Figure 1E). Seven CARs led to the non-specific proliferation of CD8 T cells. 9A8 and 1G3 did not proliferate in response to SupT1 NT (Non-Transduced), but proliferated against SupT1 $CD22^{Mid}$ (Figures 1F and S3B). Hence, 9A8 and 1G3 were taken forward for further characterization.

Lack of correlation between antibody biophysical characteristics and CAR function

We attempted to find a correlation between binding on- and off-rates, binder stability, or the identity of membrane domain (3–6) recognized. Therefore, the CAR cytotoxicity (effector to target ratio (E:T = 1:4), cytokine production (E:T = 1:4), and proliferative capacity (E:T = 1:1) against the SupT1 $CD22^{High}$ and $CD22^{Mid}$ were correlated with the biophysical properties of the binders (Figures 2A–2E).

As shown in Figure 2A, the cytotoxic function of CART did not correlate with the CD22 Ig domain recognized by the CARs, as two of the most sensitive CARs bind between the fifth and sixth Ig domains, whereas another sensitive CAR binds the third Ig domain. Similarly, efficacy failed to correlate with the affinity, with the three most efficacious binders bearing an affinity of 0.52 nM, 1.9 nM, and 36.5 nM, respectively (Figure 2D). Further, neither the association, dissociation, nor the antibody stability were correlated with lytic capacity (Figures 2B and 2C). However, the stability range of the antibodies was limited with 11 of 12 binders falling in the 67.0–71.7°C window (Figure 2E).

There was no correlation observed between the different binders and the aforementioned parameters. Comparison of 7E1 and 9A8 is illustrative; both bore very similar biophysical properties and additionally bound overlapping epitopes (Figure S1C), while their binding distance from the membrane, affinity, and stability were matched. Despite almost having no differences in their biophysical properties, their efficacy against mid-density targets was markedly different with a median target cell survival of 87.24% and 5.62% for 7E1 and 9A8, respectively. The conventional screening methods, such as three-dimensional affinity based on surface plasmon resonance, failed to predict the disparity in the function of 7E1 and 9A8. Crystallography of 7E1 and 9A8 was attempted to explore if binding angle could explain functional differences; however, this failed because of the high CD22 glycosylation.

9A8-CAR is sensitive against low-density CD22 targets

After the screening of the CD22 CARs, two candidates were selected (9A8 and 1G3) for further study. Both 9A8 and 1G3 folded as scFv as shown by efficient binding to sCD22 compared with Fab (Figure S4A), so a standard scFv architecture was used for further study.[33] 9A8 and 1G3 CAR-transduced T cells were labeled with recombinant sCD22 (Figure 3B). At this juncture, M971 CAR was also introduced as a reference given the experience with this CAR in the clinic.

Next, we carried out co-cultures to investigate the cytotoxic capacity of CARs against SupT1 $CD22^{High}$ and $CD22^{Mid}$. While CD22 target density on B-ALL is described as 2,839–3,470 molecules per cell,[12] Fry et al.[20] have described the downregulation of CD22 at the

Figure 1. Screening of novel binders recognizing CD22

(A) CD22 is a large protein bearing an ectodomain of seven Ig domains. The C2 type Ig domains are labeled 1–6, with domain 6 being the most proximal to-the-membrane domain. (B) Wistar rats or hyper-immune Abverimice were immunized against CD22 ectodomain to obtain novel binders recognizing against CD22. The biophysical properties of the binders, such as the Ig domain recognized and kinetics, are shown in (B). (C) All binders were incorporated into a retroviral cassette into a Fab CAR format and screened against the non-specific SupT1 NT cell line, or SupT1 cells engineered to express high- or mid-CD22 density. CAR T cells were challenged with 5×10^4 SupT1 NT, $CD22^{High}$, or $CD22^{Mid}$ for 72 h at a 1:4 E:T ratio. We measured the secretion of IFN-γ (D) and IL-2 (E) by ELISA. The supernatant was harvested from a co-culture of 5×10^4 target cells with CAR T cells at 1:4, 72 h after the assay execution. (F) The proliferation of CAR T cells was validated by labeling the T cells with CTV and challenging them with 5×10^4 SupT1 NT, $CD22^{High}$ and $CD22^{Mid}$ at a 1:1 ratio. The CTV dilution was measured on day 4, and the percentage of proliferation for each CAR condition was normalized to the equivalent condition in SupT1 NT for CD8 cells. One-way ANOVA was used to measure the statistical significance of the novel CARs compared to the negative FMC63 control. Comparisons between two CARs were carried out using Student's t-test (n = 8–12).

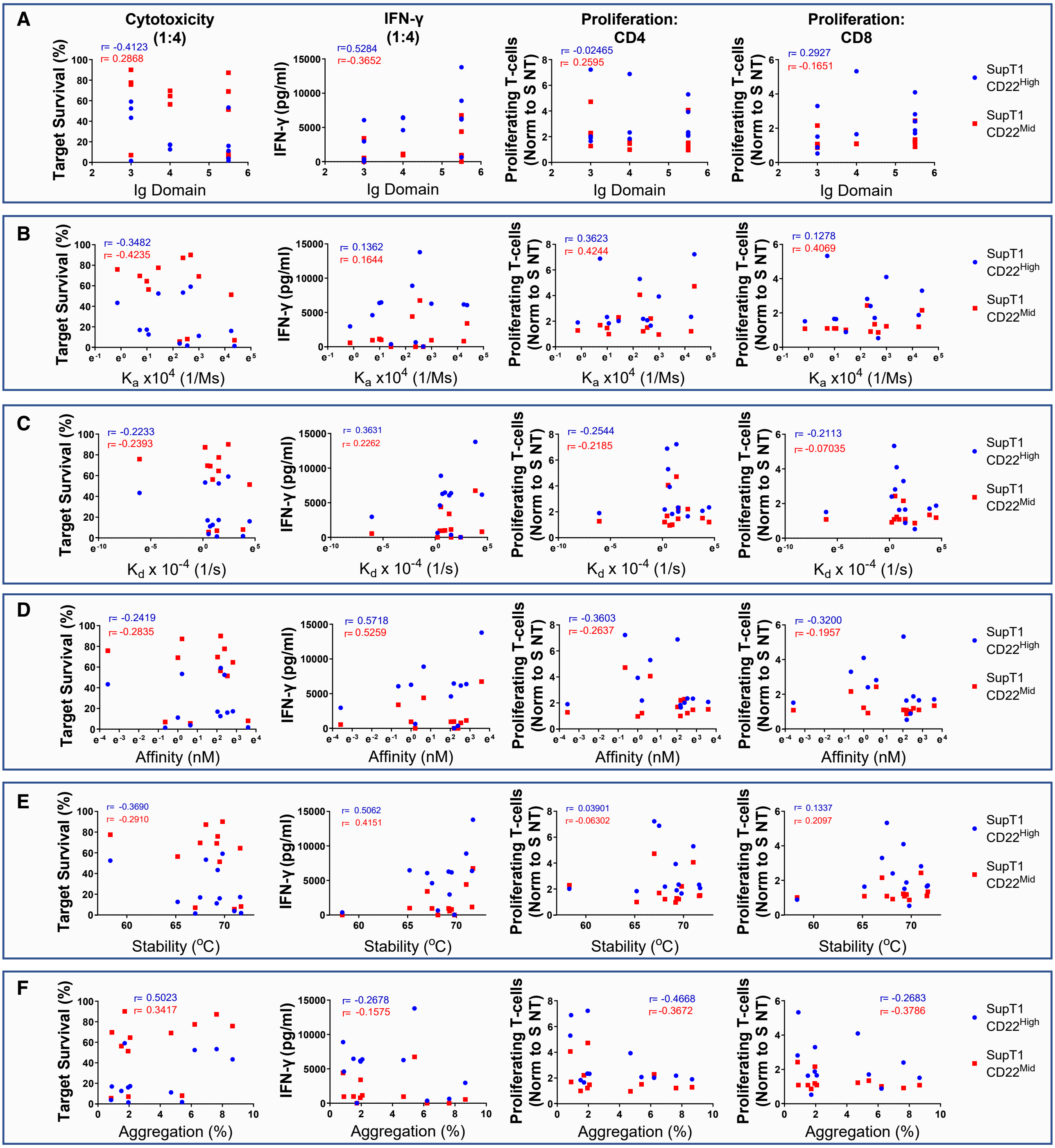

Figure 2. Parameters affecting CAR efficacy

To determine the effect of the binder biophysical properties to the CAR efficacy, we calculated two-dimensional correlation plots of Ig domain (A), association rate (K_a) (B), dissociation rate (K_d) (C), affinity (K_D) (D), binder stability (°C) (E), and aggregation (F) to the CAR efficacy. For the CAR efficacy, we introduced the cytotoxicity of the novel aCD22 binders against SupT1 $CD22^{High}$ (blue) or $CD22^{Mid}$ (red) in 1:4 ratio. The r values represent Pearson correlation coefficients (n = 8–12).

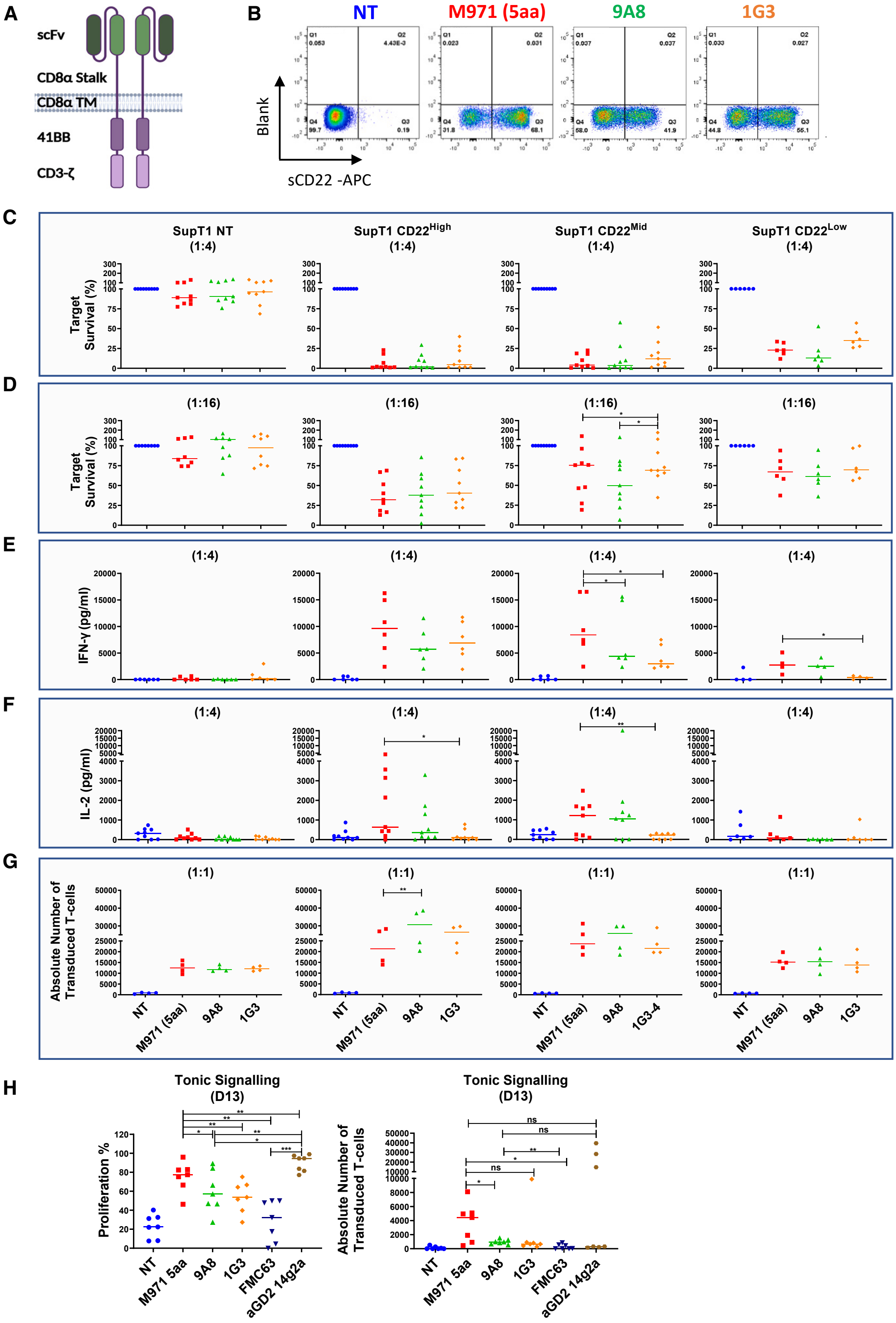

(legend on next page)

presentation of relapse. To test CART function against a lower antigen density platform, an additional SupT1 was engineered (SupT1 CD22^{Low}), where transgenic CD22 expression was constrained by a preceding inefficient stop codon so that expression was approximately 490 molecules per cell (Figure S2).[34]

All CARs tested were functional against CD22^{High}, CD22^{Mid}, and CD22^{Low} SupT1 tumor cells (Figures 3C and 3D). There was a trend of 9A8 displaying a higher sensitivity against the CD22^{Low} targets at an E:T ratio of 1:4, but despite the six donors screened, the trend did not reach significance (Figure 3C). The overall cytotoxic capacity decreased at the suboptimal ratio of 1:16 (Figure 3D), although there was a trend of 9A8 being superior against CD22^{Mid}-expressing targets. The IL-2 secretion showed no significant difference between M971 and 9A8, whereas the 1G3 IL-2-secreting function was decreased compared with M971 (Figure 3F).

The cytolytic activity of 9A8 CAR was also compared with that of LT22, another clinically proven CD22 CAR, against CD22^{Mid} and CD22^{Low} targets (Figure S3G).[26] 9A8 was superior to LT22 CAR, with higher sensitivity against both mid- and low-density target cells at a low E:T ratio.

Next, we compared the proliferative capacity of the three CARs against the SupT1 target cells (1:1). Figure 3G depicts the absolute number of transduced CART recovered after day 4 of co-culture. The proliferative capacity of 9A8 was superior to M971 when challenged with CD22^{High}- and CD22^{Mid}-expressing target cells.

Chronic basal signaling of CART in the absence of ligand, known as tonic signaling, may bestow a deleterious effect on CART efficacy and persistence.[35,36] To interrogate the 9A8 and 1G3 CARs for tonic signaling, we investigated their capacity for autonomous proliferation in the absence of antigen (Figure 3H). For comparison, we introduced the anti-CD19 FMC63-CAR as a negative control, whereas anti-GD2 14g2a was used as a positive control for tonic signaling, as it has been previously reported.[36] The T cells were labeled with CellTrace Violet and cultured in the absence of cytokines and antigenic stimulus. The exhaustion profile was comparable for M971, 9A8, 1G3, and FMC63 CARs (Figure S4E). However, the autonomous proliferation of M971 was significantly higher than the negative control FMC63, as well as 9A8 and 1G3. Both novel CD22 CARs were comparable with FMC63 in their level of tonic signaling.

To maximize the resolution of functional discrepancies, we set up a re-stimulation assay, wherein the T cells were sequentially stimulated with target cells expressing low levels of CD22 (SupT1 CD22^{Mid} and CD22^{Low}) (Figure S4F). M971 and 1G3 failed to retain the cytolytic capacity against SupT1 CD22^{Low} by Stim 3, whereas 9A8 eliminated 50.4% of CD22^{Low} cells.

An *in vivo* comparison of 9A8 with M971 was carried out in a Nalm-6 model. No significant differences were observed in tumor burden as measured by bioluminescence imaging (BLI) (Figures S5C and S5D) or flow cytometry (Figure S5E).

Of the 12 novel binders, we chose 9A8 as the binder for further study based on superior cytotoxicity against SupT1 CD22^{Low} expressing targets, as well as increased IL-2 secretion and proliferation (Figure 3).

Co-transduction with a CD19/aCD22 dual CAR targeting

A key application for CD22 CARs is combination with CD19 CARs to prevent antigen escape.[2,4,5] We next explored co-targeting CD22 and CD19 using 9A8. For CD19 targeting we used CAT-41BB-Z, a clinically proven CD19 CAR (Figure 4A).[2,29] To avoid the loss of stability or loss of expression, we used co-transduction as a strategy for co-expression (Figure 4B).

CAT/9A8 CAR T cells were generated by double transduction with two separate lentiviral vectors at a multiplicity of infection (MOI) of 2.5/2.5. CAT and 9A8 CART were generated by single transduction with an MOI of 2.5. Labeling showed distinct single and double CAR-positive populations in CAT/9A8 CAR T cells as shown in Figure 4C. The median fluorescence intensity of the CAT or 9A8 CAR in the co-transduced product was comparable with the single CARs. The CAT/9A8 CAR comprises three transduced populations at a ratio of 1:2.1:1.7 for Single^{9A8}, or Single^{CAT} or double-positive populations. The expression of the CAT or 9A8 CAR in the co-transduced product was comparable to the single CARs (Figures S6A and S6B). An anti-idiotype against CAT was used to label the CART for flow cytometry.

The effectors were challenged against SupT1 NT, SupT1 CD19^{High}, CD22^{High}, CD22^{Mid}, and CD19^{High}CD22^{High}. The cytotoxic and cytokine secretion capacity of the double transduced CAT/9A8 was comparable with the single CARs as shown in Figures 4D and 4E. Finally, all CARTs were tested against SupT1 CD22^{Mid}, wherein CAT/9A8 was not inferior to 9A8. Notably, the magnitude of cytokine response was not increased with double-positive targets (Figures 4E and S6D–S6F).

Additionally, CAT, 9A8, and CAT/9A8 CARTs were tested in co-cultures with Raji and Raji CD19^{KO} cells (Figure 4F). As expected, 9A8

Figure 3. Comparison of novel CD22 binding CARs to M971 in a lentiviral platform

(A) The best aCD22 CAR binders, 9A8 and 1G3, were transferred into a lentiviral EF1a promoter platform in an scFv format. (B) The expression of the transgenes on T cells was analyzed by flow cytometry. Specifically, T cells were labeled with biotinylated sCD22, and subsequently labeled with streptavidin-APC and Sytox Blue viability dye. A representative donor is shown in (B). The CAR cytotoxicity was measured against 1×10^5 SupT1 CD22^{High} and CD22^{Mid} target cells at 72 h and an E:T ratio of 1:4 (C) or 1:16 (D). An additional target was introduced, SupT1 CD22^{Low} engineered to express approximately 490 molecules/cell. The cytokine secretion was measured by ELISA at 72 h for 1:4 E:T. The cytokines measured were IFN-γ (E) and IL-2 (F). (G) We investigated the proliferative capacity of the CARs in a 1:1 co-culture measured by the T cell expansion by day 4. (H) CAR T cells were labeled with CTV and challenged in a starvation assay without antigen stimulus or IL-2 for 13 days to measure tonic signaling. The FMC63 and aGD2 CARs were introduced as tonic signaling negative and positive controls. The statistical significance was validated by Student's t-test. (n = 6–9).

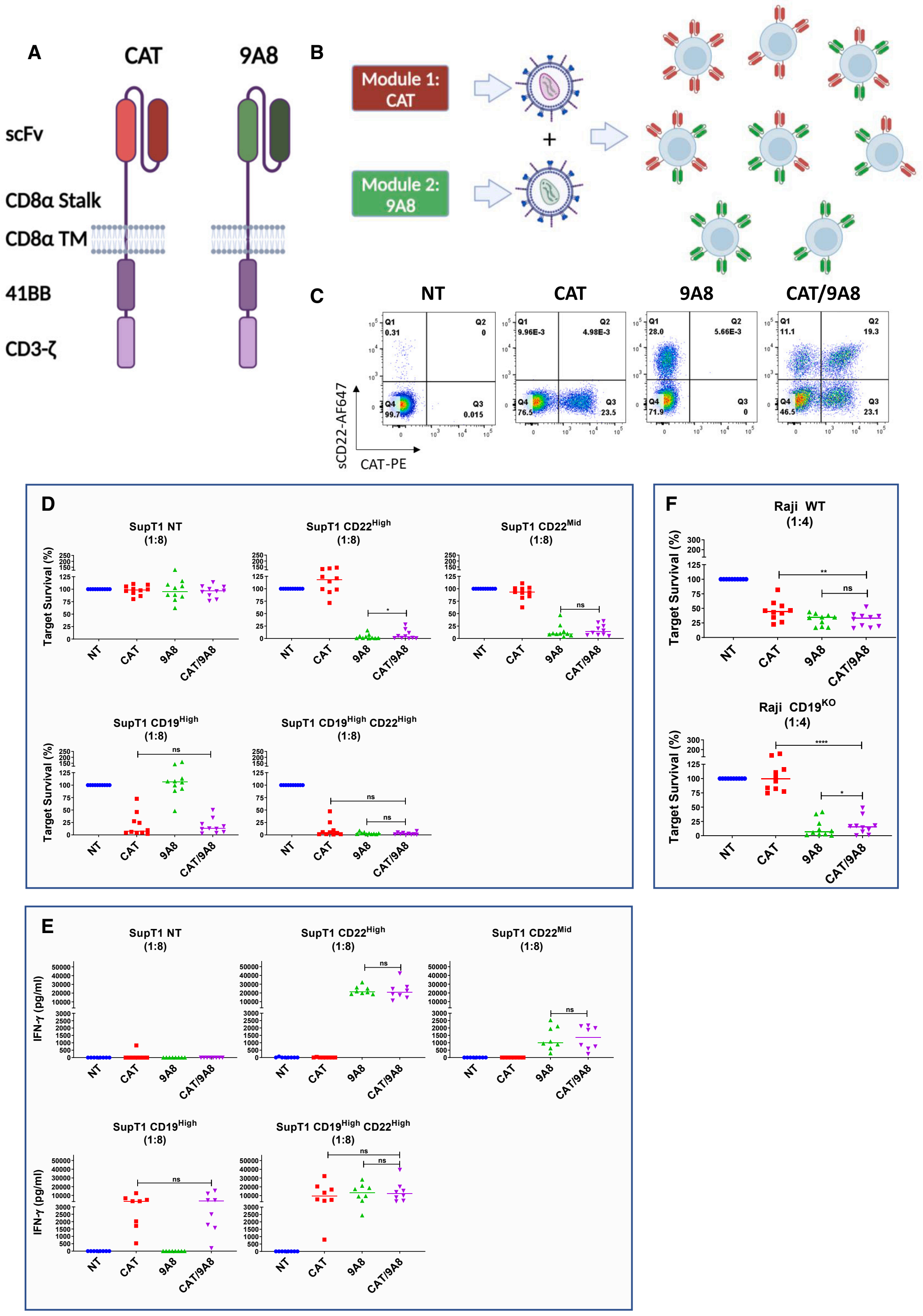

(legend on next page)

and CAT/9A8 lysed both Raji wild type (WT) and the CD19^{KO}. However, CAT was unable to eliminate Raji CD19^{KO}. CAT/9A8 was comparable to the 9A8 single transduction against both Raji WT and CD19^{KO}.

We next explored cytotoxicity against primary B-ALL samples (Figure 5). Although CAT efficiently eliminated the patient sample #20018, which was positive for both CD19 and CD22, it failed to eliminate the CD19$^{−}$ sample (P #CPL-05) (Figure 5C). In contrast, both 9A8 and CAT/9A8 conditions lysed P #CPL-05.

The cytotoxicity and cytokine secretion of CAT/9A8 *in vitro* was comparable with that of the single CARs, not compromised by the absence of CD19 or low CD22 expression, both of which constitute antigen escape mechanisms contributing to relapse in clinical studies.

CAT/9A8 is effective against a CD19 antigen escape xenograft model

We next tested CAT/9A8 CAR T cells in an *in vivo* model of B-ALL. HA-tagged Luciferase-expressing NALM-6 cells were engrafted in NSG mice. At a dose of CAR T cells established to sub-optimally eradicate disease, we compared the efficacy of CAT versus CAT/9A8 T cells over the course of 14 days. Tumor eradication was monitored by BLI (Figures 6B and 6C) or HA-tag^{+} tumor cells in the bone marrow (Figure 6D). BLI imaging showed that CAT/9A8 (3.14xe^{8}) was more efficacious at eliminating the tumor burden compared with CAT (21.3xe^{8}).

To simulate CD19 antigen escape, we genetically edited Nalm-6 cells with CRISPR/Cas9 to knockout CD19 (Figure S7). CAT and CAT/9A8 tumor eliminating efficacy was tested in a Nalm-6 CD19^{KO} model in NSG mice. CAT CAR T cells failed to eradicate the tumors. In contrast, CAT/9A8 CAR T cells, ablated the CD19$^{−}$ tumors as shown by BLI and flow cytometry (Figures 6F–6H).

CAT/9A8 CART comprise three transduced populations: Single^{CAT}, Single^{9A8}, and double-positive. The proportion of each population is shown in Figure 6I (and Figure S8). All three transduced populations were equally present before and 14 days after injection for Nalm-6 WT. In the absence of CD19 antigen, the Single^{CAT} population did not persist, whereas the expression of Single^{9A8} and double-positive were 1:1 at 44.8% and 46.1%, respectively.

The efficacy of CAT/9A8 was validated *in vivo* showing it is non-inferior to CAT CARTs in a Nalm-6 stress model. Additionally, CAT/9A8 was effective in a model of CD19-negative escape based on Nalm-6 CD19^{KO} tumor cells.

A comparison of CAT/9A8 was also done with the LoopCAR[27,37] using Nalm-6 CD19^{KO}. The LoopCAR failed to eliminate the CD19^{-} tumors in contrast to CAT/9A8 (Figure 7).

DISCUSSION

Autologous CAR T cells targeting CD19 have had considerable success in the adoptive therapy of several B-lineage malignancies.[2,29,38–43] CD19 loss is a well described mechanism of tumor escape after CD19 CAR T cell therapy.[44] Loss of CD19 surface expression is found in 30%–90% of relapsed B-ALL.[45] CD19 loss is less studied after CD19 CAR therapy in DLBCL, but is reported in 28%–62% of relapses.[46,47] A strategy to reduce target antigen escape is to target a second B lineage antigen simultaneously. CD22 is an attractive option for such a second antigen, given its expression across B cell ontogeny and consequent broad expression by B cell malignancies.

CD22 has been successfully targeted using an antibody drug conjugate.[48] However, CD22 is a challenging CAR target: its antigen density is 10-fold lower than CD19[12,49] and can down-modulate upon CAR targeting.[20] In addition, it is a tall, bulky, and heavily glycosylated protein that would be expected to resist effective approximation of T cells or target cells, preventing effective immune synapse formation.[50,51] Despite this, CD22 CAR T cells have shown efficacy against both B-ALL and DLBCL.[20,21] Notably, the receptor used in the majority of CD22 CAR T cell studies is based on the human naive phage library derived binder M971.[25]

The rules for designing a potent CAR, of particular importance to challenging targets such as CD22, seem to be established. For instance, the findings regarding the correlation between the biophysical properties of CARs and their performance weigh heavily in favor of high affinity binding domains,[52–57] with rare contradictory reports.[28,58] Similarly, several reports indicate that CARs targeting epitopes proximal to the membrane bear superior cytotoxic capacity,[12,59,60] wherein a many-fold density increase was required for membrane-distal epitopes to stimulate a response comparable to membrane-proximal ones.[61]

One possible criticism of the above work is that relatively few reports test a large set of binding domains. Through two immunization campaigns in rats or hyper-immune mice, we obtained 12 novel and unique antibodies with an affinity range of 28 pM–36.5 nM. Those

Figure 4. *In vitro* validation of CAT/9A8

(A) CAT and 9A8 were expressed on a separate lentiviral pCCL vectors driven by a PGK and EF1α promoter, respectively. (B) Lentiviral vectors produced transiently in 293T were mixed 1:1 to transduce primary T cells (MOI = 2.5 + 2.5). The CAT/9A8 product constitutes the expression of both CAT and 9A8 CARs at a stochastic mix. (C) The CAR expression was measured by the labeling of T cells with anti-CAT idiotype and sCD22 antigen as shown in x- and y axes, respectively. The mix of the vectors leads to a profile of three transduced populations of Single^{CAT}, Single^{9A8} and double-positive. We cultured 5×10^{4} SupT1 cells engineered to express CD22^{High}, CD22^{Mid}, CD19^{High}, or CD19^{High}CD22^{High} with CAR T cells at a ratio of 1:8 for 72 h. At the endpoint of this assay, the target cell lysis (D) and IFN-γ release (E) were assessed. (F) The efficacy of the CAT/9A8 product was also tested against the physiological Raji WT cells and Raji CD19^{KO}, which was implemented to simulate CD19 antigen escape. Student's t-test was utilized to determine statistical significance (n = 10).

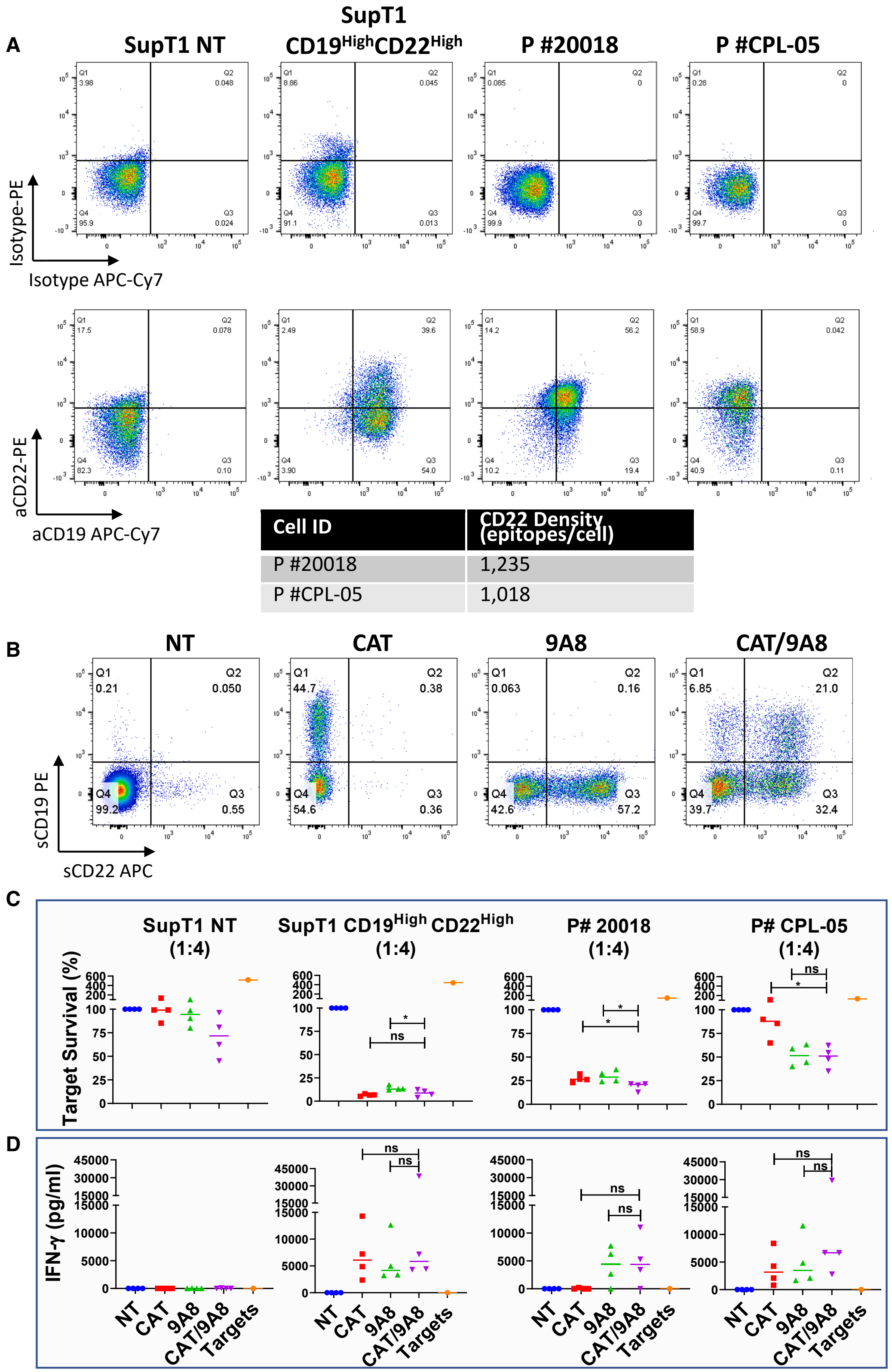

Cell ID	CD22 Density (epitopes/cell)
P #20018	1,235
P #CPL-05	1,018

(legend on next page)

12 antibodies were tested in CAR format *in vitro*. Analyses showed no correlation between the biophysical properties and *in vitro* CAR performance. This observation contrasts with the published literature, the majority of which reports a positive correlation between affinity and CAR cytotoxicity.[52–57] Similarly, we observed no correlation between CAR function and epitope distance from the membrane. However, our study was limited between the third and sixth membrane proximal Ig domains and a threshold where efficient exclusion of CD45 is achievable may exist.[51]

7E1 and 9A8 constitute a matched pair and are illustrative since their biophysical properties are almost identical, and they bind overlapping epitopes. Nevertheless, 9A8 was the most sensitive CAR and 7E1 one of the least efficacious, with a 15.5-fold lower target cell killing. One possible explanation is a difference in the binding angle of the binder latching onto CD22, thus affecting the formation of the synapse. Crystallography failed, likely because of the high glycosylation of CD22. Further analysis will be required to elucidate the underlying mechanism explaining the differential function between these two CARs.

9A8- and 1G3-based CARs were further characterized and compared with the M971 CAR. In starvation assays, 9A8 showed no tonic signaling, whereas M971 showed non-antigen-specific proliferation. M971-based CARs are known to require an unusually short linker between variable fragment heavy chain/variable fragment light chain, which may promote concatemerization, allowing high sensitivity at the cost of tonic signaling.[62] Further comparisons revealed a trend of 9A8 CART proliferating more than M971 and 1G3 CAR T cells, while showing minimal tonic signaling. Hence, we chose 9A8 for subsequent studies.

Several strategies can be used to simultaneously target CD19 and CD22 CARTs. The simplest approach is the sequential administration of anti-CD19 and anti-CD22 CAR T cells, which has been shown in clinical studies to decrease antigen escape.[63,64] For example, Pan et al.[63] demonstrated that the sequential approach is safe and achieved a 1-year leukemia-free survival rate of 79.5%. The limitation of this strategy is doubling of manufacture cost. Alternatively, dual targeting can be achieved. One approach to dual targeting is a single CAR that targets both CD19 and CD22 by bearing two antigen-binding domains. This approach is technically challenging and clinical testing of bi-specific CARs has been disappointing.[27,65,66]

A compromise between these two approaches is to co-express two separate CARs in a single T cell. The simplest way to achieve this is by use of a bicistronic cassette.[26] One report of bicistronic CD19/CD22 CAR cassette showed poor CAR T cell persistence, precluding a complete assessment of effects on antigen escape. We have previously described and clinically tested CAT-41BBζ obecabtagene autoleucel (obe-cel), a fast binding off-rate CAR designed to decrease toxicity and improve persistence.[28,29] It has been reported that bicistronic cassettes can decrease transgene expression.[67] In the present study, we explored co-transduction as means of co-expression. This approach decreases the risk of perturbation of expression of either receptor.

The efficacy of the double transduced product was tested *in vitro* showing the activity of CAT/9A8 being comparable with the single CAR controls. We tested the cytotoxic and cytokine secretion capacity of CAT/9A8 against Raji and SupT1 target cells, including $CD22^{Mid}$-expressing SupT1. CAT/9A8 consistently performed comparably with the single CARs.

An *in vitro* cytotoxicity assay against $CD19^{KO}$ Raji (1:4) showed slightly better cytotoxicity with 9A8 CAR T cell effectors in comparison with CAT/9A8. In these experiments, equal numbers of transduced T cells were used; however, not all of the CAT/9A8 CAR T cells express the 9A8 CAR and this lower 9A8 CAR transduction efficiency may explain the decreased cytotoxicity.

In vivo studies showed the efficacy of CAT/9A8 against a Nalm-6 tumor. In a Nalm6 $CD19^{KO}$ model, imitating CD19 antigen escape, CAT/9A8 eliminated the tumor burden. CAT/9A8 proved superior in a CD19 escape *in vivo* model to CD19/CD22 loop CAR.

CD22 CAR targeting is more challenging than CD19; for instance, in clinical studies relapse has been observed despite CD22 CAR T cell persistence.[20] Notably, in cytotoxicity assays against primary B-ALL blasts, CAT/9A8 and 9A8 cytotoxicity was reduced against $CD19^{-}$/$CD22^{+}$ B-ALL compared with a double-positive patient sample. This may hint at an inherent biological resistance of CD22 as a target.

Strategies that allow the co-expression of two CARs afford the freedom of using different costimulatory domains tailored to each antigen.[26,66] In fact, Shalabi et al.[66] showed the benefit of mixing co-stimulatory domains using CD19-CD28ζ and CD22-41BBζ CARs. This is worthy of future exploration.

In summary, we identified a novel CD22 targeting CAR. The process of obtaining the novel antibody revealed a lack of correlation between binder biophysical properties and the CAR function. Functional selection allowed identification of CARs sensitive to very low antigen density. A co-transduction approach was explored with 9A8-41BBζ

Figure 5. Cytotoxic efficacy of CAT/9A8 against primary B-ALL patient cells

(A) The expression of CD22 and CD19 antigens was validated by flow cytometry and CD22 density was calculated by using Quantibrite beads. (B) PBMCs were transduced with either the single CAR constructs or co-transduced with the CAT/9A8 product at an MOI of 2.5 + 2.5. We validated the transduction yield by labeling the T cells with soluble antigens. (C) The cytotoxic efficacy of the CARs against the primary B-ALL samples was measured by co-culture and subsequent target cell enumeration at 48 h after co-culture (E:T = 1:4). SupT1 NT and $CD19^{High}CD22^{High}$ constituted a negative and positive lysis control, respectively. (D) IFN-γ production was measured by ELISA at 48 h, E:T ratio 1:4. Statistical significance was determined by Student's t-test (n = 4).

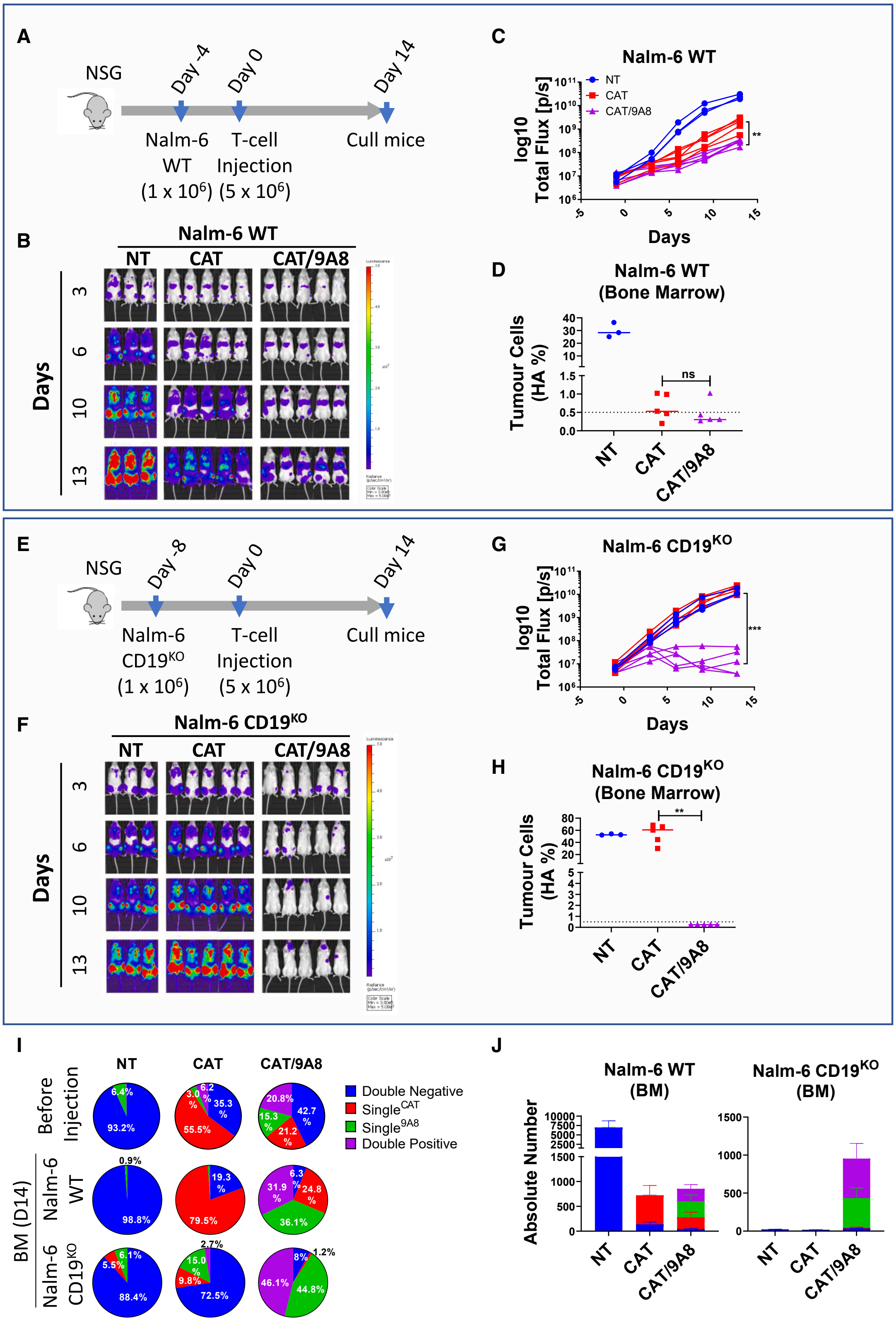

(legend on next page)

and CAT-41BBζ, resulting in a CAR T cell product effective at targeting both CD19 and CD22. Autologous T cells co-transduced with CAT/9A8 are currently being clinically tested in children with relapsed or refractory B-ALL (NCT02443831).

MATERIALS AND METHODS

Immunization campaign

Five mice (DiversimAb) were immunized with recombinant human CD22 (1968-SL, Sino Biologicals), according to proprietary protocol (Abveris). Three Wistar rats were genetically vaccinated with a truncated form of human CD22 encoding the four membrane proximal domains, according to proprietary protocol (Aldevron). Sera from immunized animals at intervals were screened for seroconversion against recombinant human CD22 via ELISA. Animals showing polyclonal reactivity against the target protein were selected for hybridoma generation.

Cell lines

HEK-293T (ATCC; ATCCCRL-11268) were cultured in Iscove's modified Dulbecco's medium (12-726F, Lonza) supplemented with 10% FBS (Labtech) and 2 mM GlutaMAX (35050061, Invitrogen). SupT1 (ECACC; 95013123), NALM6 (DSMZ; ACC 128) and Raji (ATCC, ATCC CCL-86) lines were cultured in complete RPMI (RPMI-1640, Lonza) supplemented with 10% FBS and 2 mM GlutaMAX.

Flow cytometry

Flow cytometry was performed using MACSQuant 10 and X flow cytometers (Miltenyi Biotec). Labeling was carried out at room temperature for 10 min with antibodies diluted in Cell Staining Buffer (420201, BioLegend). The antibodies used were:

aCD22 CAR was detected by sCD22 labeling (SI2-H82F8, Acro) and streptavidin-APC (405207, BioLegend); the cytotoxicity panel comprised aCD3-PeCy7 (344816, BioLegend), aCD2-PE (300208, BioLegend), aCD8-APC Cy7 (301016, BioLegend), and counting beads (C36950, Invitrogen); for the *in vivo* models we used aCD45-BV421 (304032, BioLegend), aHA-AF488 (901509, BioLegend), aCD22-PE (302506, BioLegend), 7AAD (420404, BioLegend). The CAT CAR was labeled with an anti-idiotype.

Retroviral and lentiviral production

HEK-293T cells (1.5×10^6) were transiently transfected with an RD114 envelope expression plasmid (RDF, a gift from M. Collins, University College London), and a Gag-pol expression plasmid (PeqPam-env, a gift from E. Vanin, Baylor College of Medicine), and transgene expressed in an SFG vector plasmid at a ratio of 1:1.5:1.5 (total DNA = 12.5 μg). The transfection was carried out with GeneJuice (70967-4, Millipore), according to the manufacturer's guidelines.

The lentiviral transfection differed in the plasmid composition wherein Gag-pol (plasmid #12251, Addgene, Waterdown, MA), pRSV-Rev (#244772, Addgene), VSV-G envelope (plasmid #12259 Addgene), and pCCL vector were introduced in a ratio of 2:1:1:4 (total DNA = 12.5 uμ). The lentiviral transgenes were driven by an EF1α promoter.

Cytotoxicity assay

Peripheral blood mononuclear cells (PBMCs) were isolated from whole blood by density gradient sedimentation via Ficoll. The source of PBMCs was buffy coats purchased from NHSBT (NC07). Isolated PBMCs were activated with aCD3 (130-093-387, Miltenyi Biotec)/ aCD28 antibodies (130-093-375, Miltenyi Biotec), 50 U IL-2 (Z00368-1, 2BScientific Limited) added at 24 h, and engineered by retroviral and lentiviral vectors at 48h. 0.7×10^6 PBMCs were plated on Retronectin coated 6-well plates (T100B, Takara Clonetech) and span at RT, 1,000 × *g* for 40 min.

The cytotoxicity readout was based on flow cytometry and enumerating the surviving target cells after a 72-h co-culture. At 96 h after transduction, CART were co-cultured with the targets at ratios of 1:2, 1:4, and 1:8. Target number was stable at 0.5×10^5 or 1×10^5 target cells, while T cells differed depending on the effector to target (E:T) ratio. Targets cells were enumerated 72 h after co-culture as alive ($7AAD^-$) and $CD3^-CD2^-CD8^-$. Target cell survival percentage was calculated by normalizing the number of viable target cells to that recovered from co-cultures carried out with NT T cells.

Cytotoxicity of primary cells

The primary B-ALL cells were acquired from UCL Institute of Child Health cell bank (P #CPL-05) or purchased from the Fred Hutch Hematopoietic Diseases Repository (P #20018). The B-ALL samples were thawed in gradual volume of PBS plus 1% FCS, enumerated, and co-cultured with CTV labeled CAR-expressing PBMCs for 48 h at a 1:4 ratio. The assay was carried out as described above.

Cytokine measurements

The cytokine concentrations were determined by ELISA on co-culture supernatants harvested at 72 h. We used IFN-γ and IL-2

Figure 6. *In vivo* validation of CAT/9A8

(A) The Nalm-6 WT stress model was performed on NSG mice, where 10^6 cells were intravenously injected on day −4 and 5×10^6 CAR-expressing T cells were injected on day 0 intravenously. (B) The Nalm-6 tumor line was engineered to express HA-tag, as well as luciferase that was used to estimate tumor burden by BLI. (C) This graph illustrates the luciferase levels detected in (B). The presence of tumor cells in the bone marrow was evaluated by flow cytometry detecting the HA-tag on the tumor cells (D). (E) The CD19 antigen escape was simulated by knocking out CD19 in Nalm-6 cells. One million $CD19^{KO}$ Nalm-6 cells were injected on day −8 and 5×10^6 transduced T cells were injected on day 0. The tumor burden was measured by BLI to detect luciferase on mice over the course of 13 days. (H) The percentage of HA-expressing tumor cells was also enumerated in the bone marrow compartment by flow cytometry. (I) The expression of each CAR subpopulation in each condition was validated *in vitro* before the injection into mice, as well as in the bone marrow of mice sacrificed on day 14. The percentages shown for BM at day 14 in graph (I) are based on the cell number events shown in the graph in (J). Statistical analyses were performed by Student's t-test (n = 5 mice, T cells derived from 1 donor).

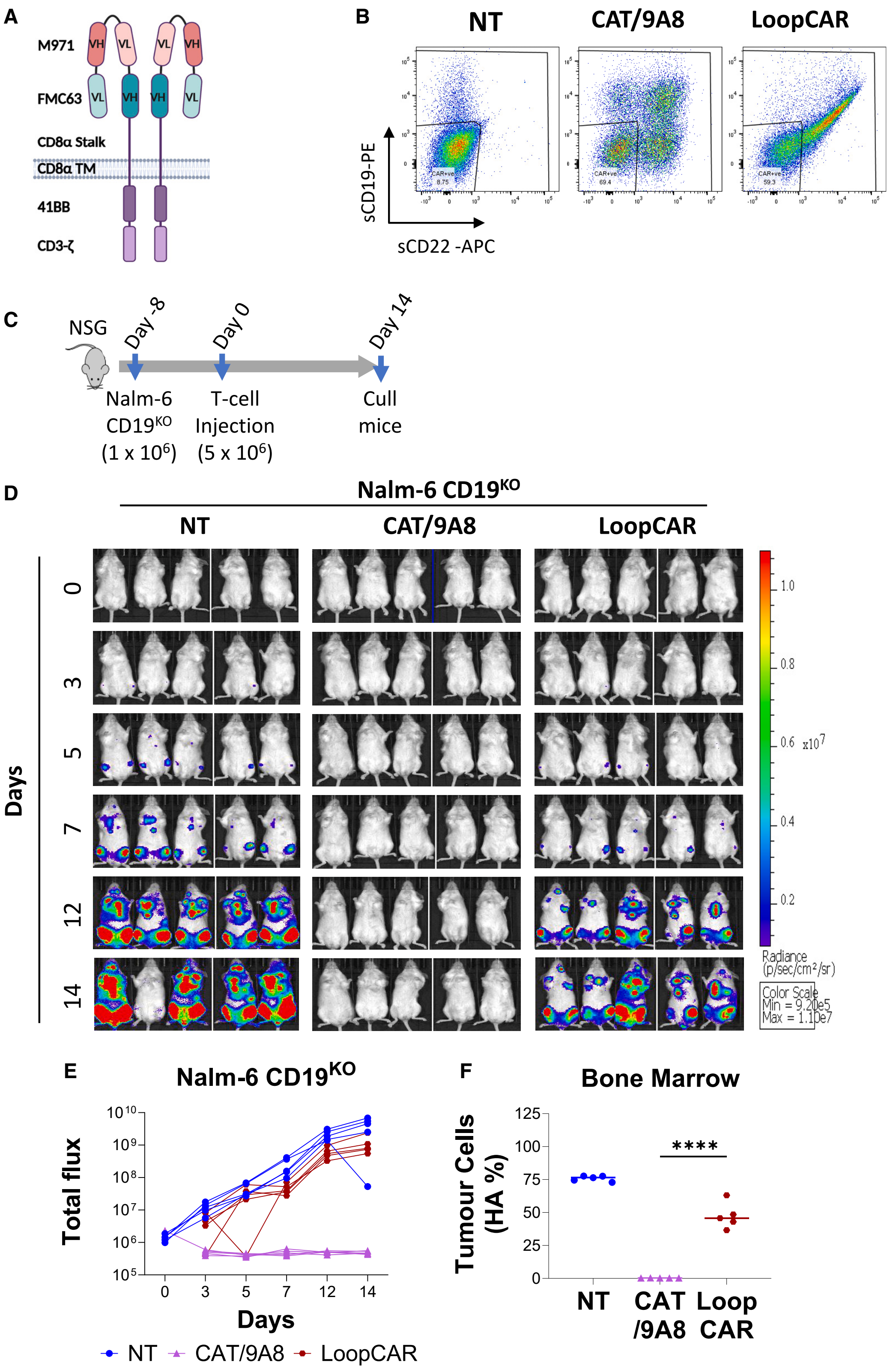

(legend on next page)

BioLegend Deluxe kits (431806 and 430106, BioLegend) according to the manufacturer's instructions.

Proliferation assay and tonic signaling

PBMCs were labeled with CellTrace Violet (CTV) according to manufacturer's instructions (C34557, ThermoFisher Scientific) and cultured with 0.5×10^5 target cells at a 1:1 E:T ratio for 96 h. The proliferation was determined as $CD3^+$ $sCD22^+$ transduced T cell number and percentage of CTV% decrease.

The tonic signaling constituted a starvation assay of 0.5×10^5 CTV-labeled T cells cultured for 13 days in the absence of targets or IL-2. The proliferation was calculated as described above.

Xenograft NSG model

All animal studies were performed under a United Kingdom Home Office-approved project license and all experiments were carried out according to the relevant regulatory standards. NSG mice (female, aged 6–10 weeks) were obtained from Charles River Laboratories and raised under pathogen-free conditions.

We inoculated 1×10^6 Nalm6 WT or $CD19^{KO}$ cells, engineered with an HA-luciferase cassette, intravenously into NSG mice on days −8 and −4, respectively. Tumor engraftment was measured by bioluminescent imaging using the IVIS spectrum system (PerkinElmer) after intra-luciferin peritoneal injection. The mice were randomized on day −1. On day 0, 5×10^6 CAR T cells were injected intravenously, and subsequently the mice were culled on day 14.

Statistical analyses

The data are visualized as median and analyses were performed in GraphPad Prism version 8.2. For a direct comparison of two conditions, we performed two-tailed paired t-test. For multiple comparisons with a single condition, we performed one-way ANOVA with Dunnett's post-test. Significance of findings are defined as follows: NS, not significant; *$p < 0.05$; **$p < 0.01$; ***$p < 0.001$; ****$p < 0.0001$.

Schematic cartoons

The cartoons were created in BioRender.

DATA AVAILABILITY

The datasets generated during and/or analyzed during the current study are available from the corresponding author on reasonable request.

SUPPLEMENTAL INFORMATION

Supplemental information can be found online at https://doi.org/10.1016/j.ymthe.2023.03.020.

ACKNOWLEDGMENTS

M.P. is supported by the University College London Hospital Biomedical Research Centre. M.P. receives salary contribution and owns stock in Autolus Ltd. E.K., S.T., S.O., and S.C. are employees of Autolus Ltd. M.P., E.K., S.P.C., S.O., S.T., B.M., and M.F. are inventors on patent WO2019/220109. M.P. is an inventor on WO2016/139487. Both patents are related to this manuscript. The primary B-ALL cells were purchased from the Fred Hutch Hematopoietic Diseases Repository or gifted from Persis Amrolia's group at UCL Child Health Institute.

AUTHOR CONTRIBUTIONS

M.P. conceived the project. S.O., B.M., and M.F. generated and characterized the binders. E.K. designed and performed the *in vitro* and *in vivo* experiments, analyzed the data and wrote the manuscript. T.G., W.H., S.M., E.C., J.T., I.G., and S.S. performed the *in vitro* experiments. L.S. and M.Robson carried out the *in vivo* work. J.S. carried out the knock-out experiments for obtaining $CD19^-$ cell lines. M.P. and S.C. reviewed the data and wrote the manuscript.

DECLARATION OF INTERESTS

Financial conflict of interest as all authors have Autolus shares. (E.K., M.F., T.G., A.B., R.J., M.Robson, J.T., M.E.K., M.Righi, J.S., I.G., S.T., and M.P. are currently Autolus employees).

REFERENCES

1. Park, J.H., Geyer, M.B., and Brentjens, R.J. (2016). CD19-targeted CAR T-cell therapeutics for hematologic malignancies: interpreting clinical outcomes to date. Blood *127*, 3312–3320. https://doi.org/10.1182/blood-2016-02-629063.

2. Ghorashian, S., Pule, M., and Amrolia, P. (2015). CD19 chimeric antigen receptor T cell therapy for haematological malignancies. Br. J. Haematol. *169*, 463–478. https://doi.org/10.1111/bjh.13340.

3. June, C.H., and Sadelain, M. (2018). Chimeric antigen receptor therapy. N. Engl. J. Med. *379*, 64–73. https://doi.org/10.1056/NEJMra1706169.

4. Sotillo, E., Barrett, D.M., Black, K.L., Bagashev, A., Oldridge, D., Wu, G., Sussman, R., Lanauze, C., Ruella, M., Gazzara, M.R., et al. (2015). Convergence of acquired mutations and alternative splicing of CD19 enables resistance to CART-19 immunotherapy. Cancer Discov. *5*, 1282–1295. https://doi.org/10.1158/2159-8290.CD-15-1020.

5. Gardner, R., Wu, D., Cherian, S., Fang, M., Hanafi, L.-A., Finney, O., Smithers, H., Jensen, M.C., Riddell, S.R., Maloney, D.G., and Turtle, C.J. (2016). Acquisition of a CD19-negative myeloid phenotype allows immune escape of MLL-rearranged B-ALL from CD19 CAR-T-cell therapy. Blood *127*, 2406–2410. https://doi.org/10.1182/blood-2015-08-665547.

6. Shalabi, H., Kraft, I.L., Wang, H.-W., Yuan, C.M., Yates, B., Delbrook, C., Zimbelman, J.D., Giller, R., Stetler-Stevenson, M., Jaffe, E.S., et al. (2018). Sequential loss of tumor surface antigens following chimeric antigen receptor T-cell therapies in diffuse large B-cell lymphoma. Haematologica *103*, e215–e218. https://doi.org/10.3324/haematol.2017.183459.

7. Moyron-Quiroz, J.E., Partida-Sánchez, S., Donís-Hernández, R., Sandoval-Montes, C., and Santos-Argumedo, L. (2002). Expression and function of CD22, a B-cell

Figure 7. *In vivo* comparison of CAT/9A8 with LoopCAR

A Schematic representation of the LoopCAR structure is shown in (A). (B) T cells bearing the LoopCAR transgene or double-transduced with CAT/9A8 were labeled with sCD19 and sCD22 to determine the transduction efficiency prior to injecting into NSG mice. (C) Nalm-6 $CD19^{KO}$ were used to simulate an antigen escape model. One million Nalm-6 $CD19^{KO}$ were inoculated in mice and CAR T cells were introduced on day 7. The tumor burden is shown by flux (D and E), whereas tumor burden in bone marrow was also measured on day 14 by labeling for the HA marker (F). (n = 5 mice, T cells derived from 1 donor).

restricted molecule. Scand. J. Immunol. *55*, 343–351. https://doi.org/10.1046/j.1365-3083.2002.01063.x.

8. Stoddart, A., Ray, R.J., and Paige, C.J. (1997). Analysis of murine CD22 during B cell development: CD22 is expressed on B cell progenitors prior to IgM. Int. Immunol. *9*, 1571–1579.

9. Boué, D.R., and LeBien, T.W. (1988). Expression and structure of CD22 in acute leukemia. Blood *71*, 1480–1486.

10. Engel, P., Wagner, N., Miller, A.S., and Tedder, T.F. (1995). Identification of the ligand-binding domains of CD22, a member of the immunoglobulin superfamily that uniquely binds a sialic acid-dependent ligand. J. Exp. Med. *181*, 1581–1586. https://doi.org/10.1084/jem.181.4.1581.

11. Huang, J., Fan, G., Zhong, Y., Gatter, K., Braziel, R., Gross, G., and Bakke, A. (2005). Diagnostic usefulness of aberrant CD22 expression in differentiating neoplastic cells of B-Cell chronic lymphoproliferative disorders from admixed benign B cells in four-color multiparameter flow cytometry. Am. J. Clin. Pathol. *123*, 826–832. https://doi.org/10.1309/KPXN-VR7X-4AME-NBBE.

12. Haso, W., Lee, D.W., Shah, N.N., Stetler-Stevenson, M., Yuan, C.M., Pastan, I.H., Dimitrov, D.S., Morgan, R.A., FitzGerald, D.J., Barrett, D.M., et al. (2013). Anti-CD22-chimeric antigen receptors targeting B-cell precursor acute lymphoblastic leukemia. Blood *121*, 1165–1174. https://doi.org/10.1182/blood-2012-06-438002.

13. Chevallier, P., Robillard, N., Houille, G., Ayari, S., Guillaume, T., Delaunay, J., Harousseau, J.-L., Avet-Loiseau, H., Mohty, M., and Garand, R. (2009). Simultaneous study of five candidate target antigens (CD20, CD22, CD33, CD52, HER2) for antibody-based immunotherapy in B-ALL: a monocentric study of 44 cases. Leukemia *23*, 806–807. https://doi.org/10.1038/leu.2008.303.

14. Piccaluga, P.P., Arpinati, M., Candoni, A., Laterza, C., Paolini, S., Gazzola, A., Sabattini, E., Visani, G., and Pileri, S.A. (2011). Surface antigens analysis reveals significant expression of candidate targets for immunotherapy in adult acute lymphoid leukemia. Leuk. Lymphoma *52*, 325–327. https://doi.org/10.3109/10428194.2010.529206.

15. Kantarjian, H., Thomas, D., Jorgensen, J., Kebriaei, P., Jabbour, E., Rytting, M., York, S., Ravandi, F., Garris, R., Kwari, M., et al. (2013). Results of inotuzumab ozogamicin, a CD22 monoclonal antibody, in refractory and relapsed acute lymphocytic leukemia. Cancer *119*, 2728–2736. https://doi.org/10.1002/cncr.28136.

16. Raetz, E.A., Cairo, M.S., Borowitz, M.J., Blaney, S.M., Krailo, M.D., Leil, T.A., Reid, J.M., Goldenberg, D.M., Wegener, W.A., Carroll, W.L., et al. (2008). Chemoimmunotherapy reinduction with epratuzumab in children with acute lymphoblastic leukemia in marrow relapse: a Children's Oncology Group Pilot Study. J. Clin. Oncol. *26*, 3756–3762. https://doi.org/10.1200/JCO.2007.15.3528.

17. Iwamoto, S., Deguchi, T., Ohta, H., Kiyokawa, N., Tsurusawa, M., Yamada, T., Takase, K., Fujimoto, J., Hanada, R., Hori, H., et al. (2011). Flow cytometric analysis of de novo acute lymphoblastic leukemia in childhood: report from the Japanese Pediatric Leukemia/Lymphoma Study Group. Int. J. Hematol. *94*, 185–192. https://doi.org/10.1007/s12185-011-0900-1.

18. Sun, Y., Wang, S.A., and Sun, T. (2018). Composite mantle cell lymphoma and chronic lymphocytic leukemia/small lymphocytic lymphoma. Cytometry B Clin. Cytom. *94*, 148–150. https://doi.org/10.1002/cyto.b.21512.

19. Olejniczak, S.H., Stewart, C.C., Donohue, K., and Czuczman, M.S. (2006). A quantitative exploration of surface antigen expression in common B-cell malignancies using flow cytometry. Immunol. Invest. *35*, 93–114. https://doi.org/10.1080/08820130500496878.

20. Fry, T.J., Shah, N.N., Orentas, R.J., Stetler-Stevenson, M., Yuan, C.M., Ramakrishna, S., Wolters, P., Martin, S., Delbrook, C., Yates, B., et al. (2018). CD22-targeted CAR T cells induce remission in B-ALL that is naive or resistant to CD19-targeted CAR immunotherapy. Nat. Med. *24*, 20–28. https://doi.org/10.1038/nm.4441.

21. Shah, N.N., Highfill, S.L., Shalabi, H., Yates, B., Jin, J., Wolters, P.L., Ombrello, A., Steinberg, S.M., Martin, S., Delbrook, C., et al. (2020). CD4/CD8 T-Cell selection affects chimeric antigen receptor (CAR) T-cell potency and toxicity: updated results from a phase I anti-CD22 CAR T-cell trial. J. Clin. Oncol. *38*, 1938–1950. https://doi.org/10.1200/JCO.19.03279.

22. Ereño-Orbea, J., Sicard, T., Cui, H., Mazhab-Jafari, M.T., Benlekbir, S., Guarné, A., Rubinstein, J.L., and Julien, J.-P. (2017). Molecular basis of human CD22 function and therapeutic targeting. Nat. Commun. *8*, 764. https://doi.org/10.1038/s41467-017-00836-6.

23. Wilson, G.L., Fox, C.H., Fauci, A.S., and Kehrl, J.H. (1991). cDNA cloning of the B cell membrane protein CD22: a mediator of B-B cell interactions. J. Exp. Med. *173*, 137–146. https://doi.org/10.1084/jem.173.1.137.

24. Dong, R., Libby, K.A., Blaeschke, F., Fuchs, W., Marson, A., Vale, R.D., and Su, X. (2020). Rewired signaling network in T cells expressing the chimeric antigen receptor (CAR). EMBO J. *39*, e104730. https://doi.org/10.15252/embj.2020104730.

25. Xiao, X., Ho, M., Zhu, Z., Pastan, I., and Dimitrov, D.S. (2009). Identification and characterization of fully human anti-CD22 monoclonal antibodies. MAbs *1*, 297–303.

26. Cordoba, S., Onuoha, S., Thomas, S., Pignataro, D.S., Hough, R., Ghorashian, S., Vora, A., Bonney, D., Veys, P., Rao, K., et al. (2021). CAR T cells with dual targeting of CD19 and CD22 in pediatric and young adult patients with relapsed or refractory B cell acute lymphoblastic leukemia: a phase 1 trial. Nat. Med. *27*, 1797–1805. https://doi.org/10.1038/s41591-021-01497-1.

27. Spiegel, J.Y., Patel, S., Muffly, L., Hossain, N.M., Oak, J., Baird, J.H., Frank, M.J., Shiraz, P., Sahaf, B., Craig, J., et al. (2021). CAR T cells with dual targeting of CD19 and CD22 in adult patients with recurrent or refractory B cell malignancies: a phase 1 trial. Nat. Med. *27*, 1419–1431. https://doi.org/10.1038/s41591-021-01436-0.

28. Ghorashian, S., Kramer, A.M., Onuoha, S., Wright, G., Bartram, J., Richardson, R., Albon, S.J., Casanovas-Company, J., Castro, F., Popova, B., et al. (2019). Enhanced CAR T cell expansion and prolonged persistence in pediatric patients with ALL treated with a low-affinity CD19 CAR. Nat. Med. *25*, 1408–1414. https://doi.org/10.1038/s41591-019-0549-5.

29. Roddie, C., Dias, J., O'Reilly, M.A., Abbasian, M., Cadinanos-Garai, A., Vispute, K., Bosshard-Carter, L., Mitsikakou, M., Mehra, V., Roddy, H., et al. (2021). Durable responses and low toxicity after fast off-rate CD19 chimeric antigen receptor-T therapy in adults with relapsed or refractory B-cell acute lymphoblastic leukemia. J. Clin. Oncol. *39*, 3352–3363. https://doi.org/10.1200/JCO.21.00917.

30. Mullen, T.E., Abdullah, R., Boucher, J., Brousseau, A.S., Dasuri, N.K., Ditto, N.T., Doucette, A.M., Emery, C., Gabriel, J., Greamo, B., et al. (2021). Accelerated antibody discovery targeting the SARS-CoV-2 spike protein for COVID-19 therapeutic potential. Antib. Ther. *4*, 185–196. https://doi.org/10.1093/abt/tbab018.

31. Oettgen, H.F., Binaghi, R.A., and Benacerraf, B. (1966). The effect of various hormones on the serum level of anaphylactic antibody in the rat. Int. Arch. Allergy Appl. Immunol. *29*, 131–139. https://doi.org/10.1159/000229694.

32. Nolan, K.F., Yun, C.-O., Akamatsu, Y., Murphy, J.C., Leung, S.-O., Beecham, E.J., and Junghans, R.P. (1999). Bypassing immunization: optimized design of "designer T cells" against carcinoembryonic antigen (CEA)-expressing tumors, and lack of suppression by soluble CEA. Clin. Cancer Res. *5*, 3928–3941.

33. Imai, C., Mihara, K., Andreansky, M., Nicholson, I.C., Pui, C.-H., Geiger, T.L., and Campana, D. (2004). Chimeric receptors with 4-1BB signaling capacity provoke potent cytotoxicity against acute lymphoblastic leukemia. Leukemia *18*, 676–684. https://doi.org/10.1038/sj.leu.2403302.

34. Sillibourne, J.E., Agliardi, G., Righi, M., Smetanova, K., Rowley, G., Speller, S., Dolor, A., Lamb, K., Allen, C., Karattil, R., et al. (2022). A compact and simple method of achieving differential transgene expression by exploiting translational readthrough. Biotechniques *72*, 143–154. https://doi.org/10.2144/btn-2021-0079.

35. Frigault, M.J., Lee, J., Basil, M.C., Carpenito, C., Motohashi, S., Scholler, J., Kawalekar, O.U., Guedan, S., McGettigan, S.E., Posey, A.D., et al. (2015). Identification of chimeric antigen receptors that mediate constitutive or inducible proliferation of T cells. Cancer Immunol. Res. *3*, 356–367. https://doi.org/10.1158/2326-6066.CIR-14-0186.

36. Long, A.H., Haso, W.M., Shern, J.F., Wanhainen, K.M., Murgai, M., Ingaramo, M., Smith, J.P., Walker, A.J., Kohler, M.E., Venkateshwara, V.R., et al. (2015). 4-1BB costimulation ameliorates T cell exhaustion induced by tonic signaling of chimeric antigen receptors. Nat. Med. *21*, 581–590. https://doi.org/10.1038/nm.3838.

37. Qin, H., Ramakrishna, S., Nguyen, S., Fountaine, T.J., Ponduri, A., Stetler-Stevenson, M., Yuan, C.M., Haso, W., Shern, J.F., Shah, N.N., and Fry, T.J. (2018). Preclinical development of bivalent chimeric antigen receptors targeting both CD19 and CD22. Mol. Ther. Oncolytics *11*, 127–137. https://doi.org/10.1016/j.omto.2018.10.006.

38. Kalos, M., Levine, B.L., Porter, D.L., Katz, S., Grupp, S.A., Bagg, A., and June, C.H. (2011). T cells with chimeric antigen receptors have potent antitumor effects and can establish memory in patients with advanced leukemia. Sci. Transl. Med. *3*, 95ra73. https://doi.org/10.1126/scitranslmed.3002842.

39. Brentjens, R.J., Davila, M.L., Riviere, I., Park, J., Wang, X., Cowell, L.G., Bartido, S., Stefanski, J., Taylor, C., Olszewska, M., et al. (2013). CD19-Targeted T cells rapidly induce molecular remissions in adults with chemotherapy-refractory acute lymphoblastic leukemia. Sci. Transl. Med. *5*, 177ra38. https://doi.org/10.1126/scitranslmed.3005930.

40. Grupp, S.A., Maude, S.L., Shaw, P.A., Aplenc, R., Barrett, D.M., Callahan, C., Lacey, S.F., Levine, B.L., Melenhorst, J.J., Motley, L., et al. (2015). Durable remissions in children with relapsed/refractory ALL treated with T cells engineered with a CD19-targeted chimeric antigen receptor (CTL019). Blood *126*, 681.

41. Davila, M.L., Riviere, I., Wang, X., Bartido, S., Park, J., Curran, K., Chung, S.S., Stefanski, J., Borquez-Ojeda, O., Olszewska, M., et al. (2014). Efficacy and toxicity management of 19-28z CAR T cell therapy in B cell acute lymphoblastic leukemia. Sci. Transl. Med. *6*, 224ra25. https://doi.org/10.1126/scitranslmed.3008226.

42. Maude, S.L., Frey, N., Shaw, P.A., Aplenc, R., Barrett, D.M., Bunin, N.J., Chew, A., Gonzalez, V.E., Zheng, Z., Lacey, S.F., et al. (2014). Chimeric antigen receptor T cells for sustained remissions in leukemia. N. Engl. J. Med. *371*, 1507–1517. https://doi.org/10.1056/NEJMoa1407222.

43. Lee, D.W., Kochenderfer, J.N., Stetler-Stevenson, M., Cui, Y.K., Delbrook, C., Feldman, S.A., Fry, T.J., Orentas, R., Sabatino, M., Shah, N.N., et al. (2015). T cells expressing CD19 chimeric antigen receptors for acute lymphoblastic leukaemia in children and young adults: a phase 1 dose-escalation trial. Lancet *385*, 517–528. https://doi.org/10.1016/S0140-6736(14)61403-3.

44. Orlando, E.J., Han, X., Tribouley, C., Wood, P.A., Leary, R.J., Riester, M., Levine, J.E., Qayed, M., Grupp, S.A., Boyer, M., et al. (2018). Genetic mechanisms of target antigen loss in CAR19 therapy of acute lymphoblastic leukemia. Nat. Med. *24*, 1504–1506. https://doi.org/10.1038/s41591-018-0146-z.

45. Majzner, R.G., and Mackall, C.L. (2018). Tumor antigen escape from CAR T-cell therapy. Cancer Discov. *8*, 1219–1226. https://doi.org/10.1158/2159-8290.CD-18-0442.

46. Plaks, V., Rossi, J.M., Chou, J., Wang, L., Poddar, S., Han, G., Wang, Z., Kuang, S.-Q., Chu, F., Davis, R.E., et al. (2021). CD19 target evasion as a mechanism of relapse in large B-cell lymphoma treated with axicabtagene ciloleucel. Blood *138*, 1081–1085. https://doi.org/10.1182/blood.2021010930.

47. Oak, J., Spiegel, J.Y., Sahaf, B., Natkunam, Y., Long, S.R., Hossain, N., Mackall, C.L., Kong, K.A., and Miklos, D.B. (2018). Target antigen downregulation and other mechanisms of failure after axicabtagene ciloleucel (CAR19) therapy. Blood *132*, 4656. https://doi.org/10.1182/blood-2018-99-120206.

48. DiJoseph, J.F., Popplewell, A., Tickle, S., Ladyman, H., Lawson, A., Kunz, A., Khandke, K., Armellino, D.C., Boghaert, E.R., Hamann, P., et al. (2005). Antibody-targeted chemotherapy of B-cell lymphoma using calicheamicin conjugated to murine or humanized antibody against CD22. Cancer Immunol. Cancer Immunol. Immunother. *54*, 11–24. https://doi.org/10.1007/s00262-004-0572-2.

49. Majzner, R.G., Rietberg, S.P., Sotillo, E., Dong, R., Vachharajani, V.T., Labanieh, L., Myklebust, J.H., Kadapakkam, M., Weber, E.W., Tousley, A.M., et al. (2020). Tuning the antigen density requirement for CAR T-cell activity. Cancer Discov. *10*, 702–723. https://doi.org/10.1158/2159-8290.CD-19-0945.

50. Anton van der Merwe, P., Davis, S.J., Shaw, A.S., and Dustin, M.L. (2000). Cytoskeletal polarization and redistribution of cell-surface molecules during T cell antigen recognition. Semin. Immunol. *12*, 5–21. https://doi.org/10.1006/smim.2000.0203.

51. Xiao, Q., Zhang, X., Tu, L., Cao, J., Hinrichs, C.S., and Su, X. (2022). Size-dependent activation of CAR-T cells. Sci. Immunol. *7*, eabl3995. https://doi.org/10.1126/sciimmunol.abl3995.

52. Chames, P., Willemsen, R.A., Rojas, G., Dieckmann, D., Rem, L., Schuler, G., Bolhuis, R.L., and Hoogenboom, H.R. (2002). TCR-like human antibodies expressed on human CTLs mediate antibody affinity-dependent cytolytic activity. J. Immunol. *169*, 1110–1118.

53. Chmielewski, M., Hombach, A., Heuser, C., Adams, G.P., and Abken, H. (2004). T cell activation by antibody-like immunoreceptors: increase in affinity of the single-chain fragment domain above threshold does not increase T cell activation against antigen-positive target cells but decreases selectivity. J. Immunol. *173*, 7647–7653. https://doi.org/10.4049/jimmunol.173.12.7647.

54. Hudecek, M., Lupo-Stanghellini, M.-T., Kosasih, P.L., Sommermeyer, D., Jensen, M.C., Rader, C., and Riddell, S.R. (2013). Receptor affinity and extracellular domain modifications affect tumor recognition by ROR1-specific chimeric antigen receptor T cells. Clin. Cancer Res. *19*, 3153–3164. https://doi.org/10.1158/1078-0432.CCR-13-0330.

55. Caruso, H.G., Hurton, L.V., Najjar, A., Rushworth, D., Ang, S., Olivares, S., Mi, T., Switzer, K., Singh, H., Huls, H., et al. (2015). Tuning sensitivity of CAR to EGFR density limits recognition of normal tissue while maintaining potent antitumor activity. Cancer Res. *75*, 3505–3518. https://doi.org/10.1158/0008-5472.CAN-15-0139.

56. Liu, X., Jiang, S., Fang, C., Yang, S., Olalere, D., Pequignot, E.C., Cogdill, A.P., Li, N., Ramones, M., Granda, B., et al. (2015). Affinity-tuned ErbB2 or EGFR chimeric antigen receptor T cells exhibit an increased therapeutic index against tumors in mice. Cancer Res. *75*, 3596–3607. https://doi.org/10.1158/0008-5472.CAN-15-0159.

57. Lynn, R.C., Feng, Y., Schutsky, K., Poussin, M., Kalota, A., Dimitrov, D.S., and Powell, D.J., Jr. (2016). High-affinity FRβ-specific CAR T cells eradicate AML and normal myeloid lineage without HSC toxicity. Leukemia *30*, 1355–1364. https://doi.org/10.1038/leu.2016.35.

58. Turatti, F., Figini, M., Balladore, E., Alberti, P., Casalini, P., Marks, J.D., Canevari, S., and Mezzanzanica, D. (2007). Redirected activity of human antitumor chimeric immune receptors is governed by antigen and receptor expression levels and affinity of interaction. J. Immunother. *30*, 684–693. https://doi.org/10.1097/CJI.0b013e3180de5d90.

59. Hombach, A.A., Schildgen, V., Heuser, C., Finnern, R., Gilham, D.E., and Abken, H. (2007). T cell activation by antibody-like immunoreceptors: the position of the binding epitope within the target molecule determines the efficiency of activation of redirected T cells. J. Immunol. *178*, 4650–4657. https://doi.org/10.4049/jimmunol.178.7.4650.

60. Wilkie, S., Picco, G., Foster, J., Davies, D.M., Julien, S., Cooper, L., Arif, S., Mather, S.J., Taylor-Papadimitriou, J., Burchell, J.M., and Maher, J. (2008). Retargeting of human T cells to tumor-associated MUC1: the evolution of a chimeric antigen receptor. J. Immunol. *180*, 4901–4909. https://doi.org/10.4049/jimmunol.180.7.4901.

61. James, S.E., Greenberg, P.D., Jensen, M.C., Lin, Y., Wang, J., Till, B.G., Raubitschek, A.A., Forman, S.J., and Press, O.W. (2008). Antigen sensitivity of CD22-specific chimeric TCR is modulated by target epitope distance from the cell membrane. J. Immunol. *180*, 7028–7038. https://doi.org/10.4049/jimmunol.180.10.7028.

62. Singh, N., Frey, N.V., Engels, B., Barrett, D.M., Shestova, O., Ravikumar, P., Cummins, K.D., Lee, Y.G., Pajarillo, R., Chun, I., et al. (2021). Antigen-independent activation enhances the efficacy of 41BB co-stimulated CD22 CAR T cells. Nat. Med. *27*, 842–850. https://doi.org/10.1038/s41591-021-01326-5.

63. Pan, J., Zuo, S., Deng, B., Xu, X., Li, C., Zheng, Q., Ling, Z., Song, W., Xu, J., Duan, J., et al. (2020). Sequential CD19-22 CAR T therapy induces sustained remission in children with r/r B-ALL. Blood *135*, 387–391. https://doi.org/10.1182/blood.2019003293.

64. Wang, N., Hu, X., Cao, W., Li, C., Xiao, Y., Cao, Y., Gu, C., Zhang, S., Chen, L., Cheng, J., et al. (2020). Efficacy and safety of CAR19/22 T-cell cocktail therapy in patients with refractory/relapsed B-cell malignancies. Blood *135*, 17–27. https://doi.org/10.1182/blood.2019000017.

65. Hossain, N., Sahaf, B., Abramian, M., Spiegel, J.Y., Kong, K., Kim, S., Mavroukakis, S., Oak, J., Natkunam, Y., Meyer, E.H., et al. (2018). Phase I experience with a Bi-specific CAR targeting CD19 and CD22 in adults with B-cell malignancies. Blood *132*, 490. https://doi.org/10.1182/blood-2018-99-110142.

66. Shalabi, H., Qin, H., Su, A., Yates, B., Wolters, P.L., Steinberg, S.M., Ligon, J.A., Silbert, S., DéDé, K., Benzaoui, M., et al. (2022). CD19/22 CAR T cells in children and young adults with B-ALL: phase 1 results and development of a novel bicistronic CAR. Blood *140*, 451–463. https://doi.org/10.1182/blood.2022015795.

67. Kumar, M., Keller, B., Makalou, N., and Sutton, R.E. (2001). Systematic determination of the packaging limit of lentiviral vectors. Hum. Gene Ther. *12*, 1893–1905. https://doi.org/10.1089/104303401753153947.

YMTHE, Volume *31*

Supplemental Information

Dual targeting of CD19 and CD22 against B-ALL using a novel high-sensitivity aCD22 CAR

Evangelia Kokalaki, Biao Ma, Mathieu Ferrari, Thomas Grothier, Warren Hazelton, Somayya Manzoor, Eren Costu, Julia Taylor, Anna Bulek, Saket Srivastava, Isaac Gannon, Ram Jha, Rosalind Gealy, Lukas Stanczuk, Tatiana Rizou, Mathew Robson, Mohamed El-Kholy, Vania Baldan, Matteo Righi, James Sillibourne, Simon Thomas, Shimobi Onuoha, Shaun Cordoba, and Martin Pule

Supplemental Methods

Surface plasmon resonance

Recombinant anti-CD22 antibodies in IgG format, were captured on flow cells 2, 3 and 4 on a Series S Protein A sensor chip (Cytiva 29650263) to a density of 40-60 RU using a Biacore T200 instrument. HBS-P+ buffer was used as running buffer is all experimental conditions. Recombinant purified CD22 (Acro Biosystems SI2-H5228) at known concentrations was used as the 'analyte' and injected over the respective flow cells with 150 s contact time and 300s dissociation at 30 µl/minute of flow rate with a constant temperature of 25°C. In each experiment, flow cell 1 was unmodified and used for reference subtraction. A '0 concentration' sensogram of buffer alone was used as a double reference subtraction to factor for drift. Data were fit to a 1:1 Langmuir binding model. Since a capture system was used, a local Rmax parameter was used for the data fitting in each case.

Epitope binning

Recombinant anti-CD22 antibodies were immobilized on flow cell 2 of channels 1, 3, 5 and 7 of a Series S CM5 sensor chip (Cytiva 29104988) to a density of 820-1350 RU using a Biacore 8k instrument. HBS-P+ buffer was used as running buffer is all experimental conditions. Recombinant purified CD22 (Acro Biosystems SI2-H5228) at 200 nM (analyte 1) was injected over the flow channels for 150s at 30 µl/min, followed by 100 nM of the challenging anti-CD22 antibody (analyte 2) for 150s at 30 µl/min. A dissociation phase of 300s was included at the end. A condition without analyte 2 injection was used as baseline to assess analyte 2 contribution to RU. A '0 concentration' sensogram of buffer alone was used as reference subtraction to factor for drift.

Cell line engineering with STOP/SKIP

In order to obtain low CD22 expressing cells, we introduced a STOP/SKIP sequence upstream of the transgene. A STOPSKIP motif is a DNA sequence that bears a stop codon followed by a read-through sequence (e.g. CATG), with the aim of reducing the translational efficiency of any downstream transgene (Loughran et al. 2014). Different STOP codons led to different expression stringency and thus expression levels.

Antigen density measurement

Antigen density was determined by labelling the cell lines with aCD22 (BioLegend; 302506), aCD19 (BioLegend; 302208), or isotype (BioLegend; 400112) antibodies conjugated to PE. Concomitantly, we ran Quantibrite beads (BD Biosciences; 10626384), based on the beads' fluorescence and know density we created a standard curve. For each sample, the median fluorescence for PE was measured and their density calculated based on the Quantibrite standard curve.

Exhaustion phenotyping

Exhaustion phenotyping was performed by labelling transduced T cells for Tim3 (BioLegend; 345008), Lag-3 (Enzo; ALX-804-806F-C100), and PD-1 (BioLegned; 621608) exhaustion markers. $0.5x10^5$ T cells were cultured at a starvation assay in the absence of antigenic stimulus or IL-2 for 6 days. The exhaustion severity was measured by the amount of markers expressed on the T cells (single, double, triple).

Supplemental Methods

Restimulation Assay

We set-up a co-culture of $5x10^4$ effectors in the presence of $5x10^4$ targets (SupT1 NT, SupT1 $CD22^{Mid}$, or $CD22^{Low}$). Fresh targets were introduced every 4-6 days for stimulation 2 and 3. Stimulation 1 (Stim 1) was measured on day 3 after the assay set-up. The lysis has been normalised to that of SupT1 NT for each time-point.

Engineering Nalm6 $CD19^{KO}$

Nalm-6 cells (DSMZ clone ACC 128) were transduced with retrovirus encoding blasticidin S deaminase and Streptococcus pyogenes Cas9. Selection for transduced cells was carried out by culturing the cells in 20 mg/mL blasticidin (Invivogen) for 2 weeks. To generate a CD22 knockout, 1x106 Cas9-expressing Nalm-6 cells were nucleofected, using SF buffer and pulse code CV104 (Lonza), with 100 pmol of sgRNA (IDT) targeting the CD22 gene (5'-GAAACCCTCTACGCCTGGGA-3'). Single cell clones were established and then transduced with retrovirus encoding the sort select marker RQR8 and chimeric CD22/CD19, consisting of the CD22 ectodomain fused to the CD19 transmembrane and truncated cytoplasmic domain (residues 333-556 deleted). These cells were nucleofected with sgRNA (IDT) targeting the CD19 gene (5'-TGGAATGTTTCGGACCTAGG-3') to generate CD19 knockout cells.

9A8 Sequence

aCD22_9A8_VK:
DIQMTQSPSSLSASLGDRVTITCRSSQDIGNYLTWFQQKVGRSPRRMIYGAIKLEDGVPSRFSGSRSGSDYSLTISSLESEDVADYQCLQSIQYPFTFGSGTKLEIKR
aCD22_9A8_VH:
EVQLVESGGGLVQPGRSLKLSCAASGFTFSNFAMAWVRQPPTKGLEWVASISTGGGNTYYRDSVKGRFTISRDDAKNTQYLQMDSLRSEDTATYYCARQRNYYDGSYDYEGYTMDAWGQGTSVTVS

References

Loughran, G., Chou, M.-Y., Ivanov, I.P., Jungreis, I., Kellis, M., Kiran, A.M., Baranov, P.V., and Atkins, J.F. (2014). Evidence of efficient stop codon readthrough in four mammalian genes. Nucleic Acids Res. *42*, 8928–8938.

Figure S1: Novel aCD22 binders and their biophysical properties

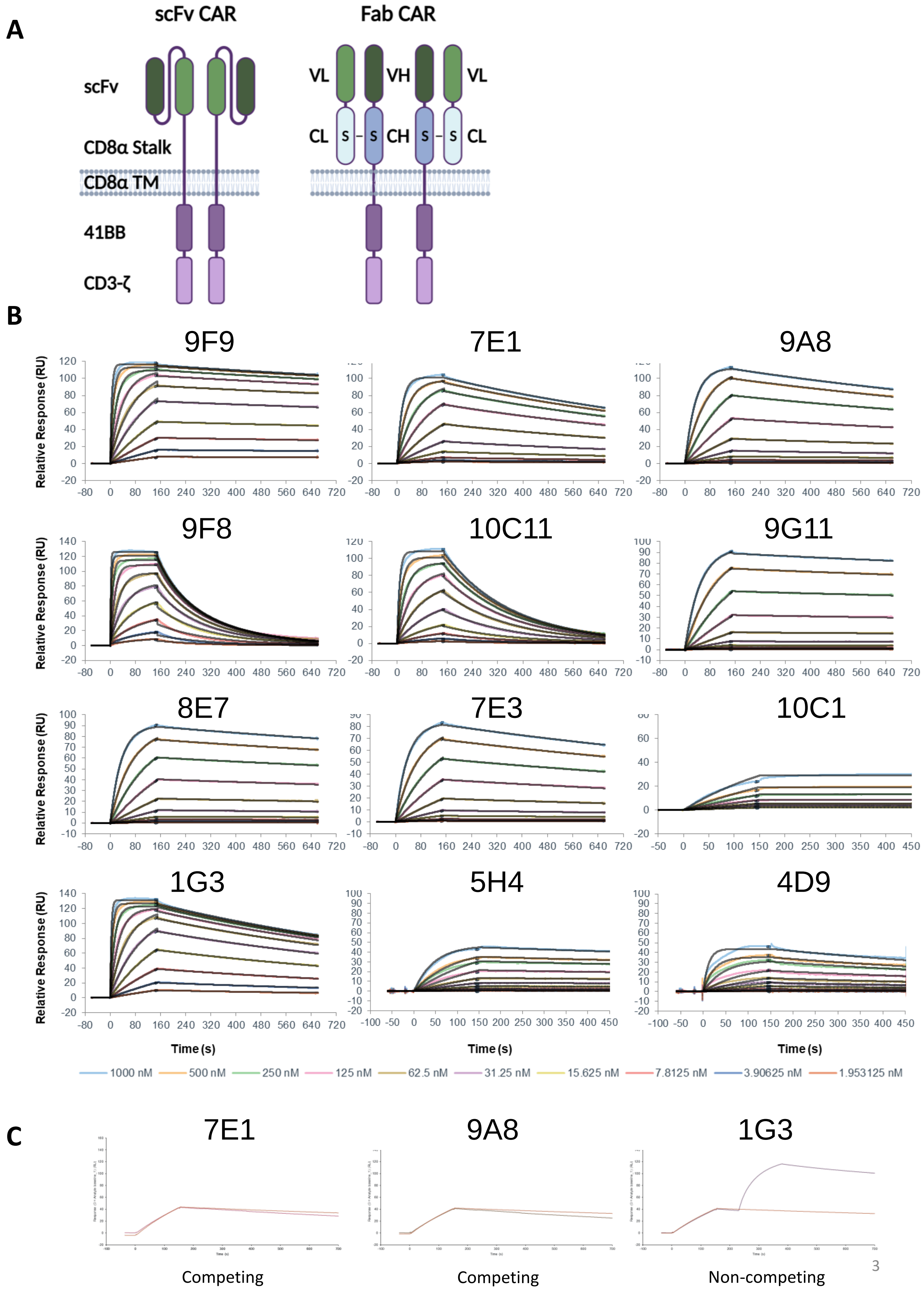

Figure S1: Novel aCD22 binders and their biophysical properties

A) The novel aCD22 CARs were screened into a Fab format, wherein the heavy and light chains were expressed separately. The light chain was fused to the IgK constant kappa domain, while the heavy chain was fused to the constant heavy chain of IgG followed by CD28 transmembrane, 4-1BB and CD3ζ. **B)** The kinetics of the binders were determined with Biacore technology. **C)** Epitope mapping was performed utilising 9A8.

Figure S2: Engineered SupT1 cells to express high and low densities of CD22

A

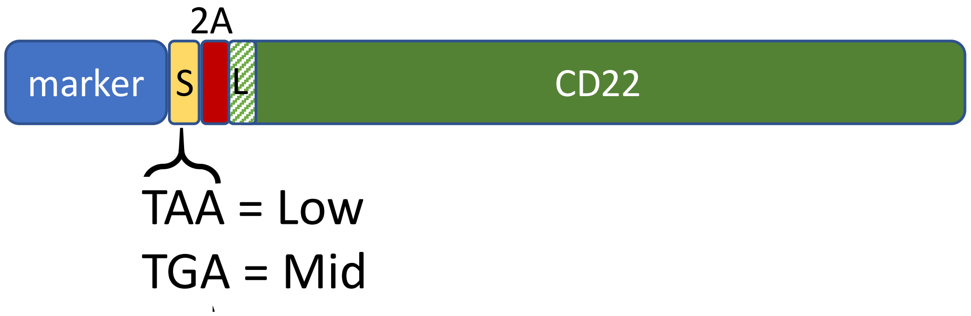

B

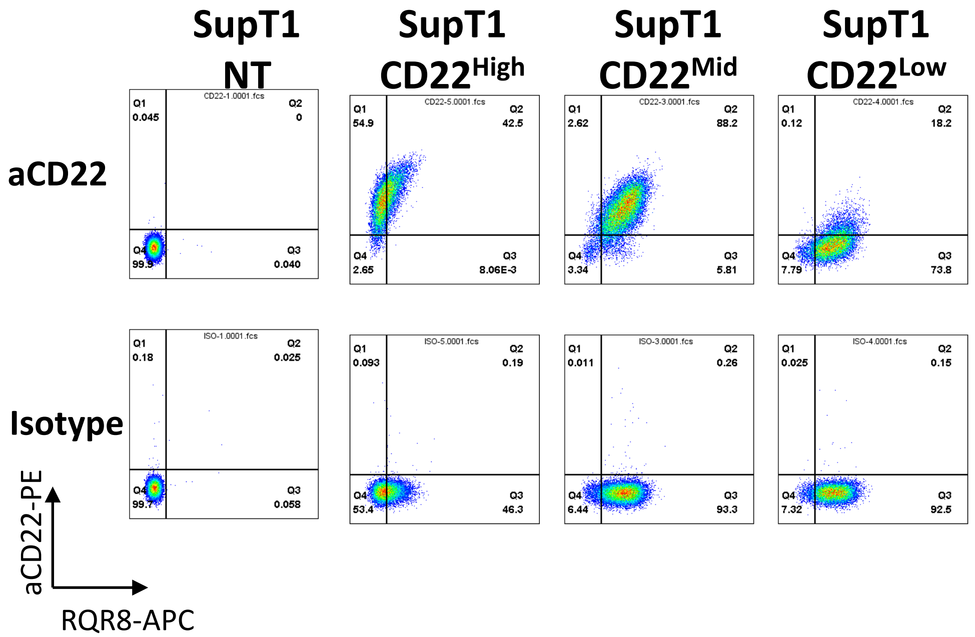

C

Cell Line	Density (Molecules / cell)
SupT1 CD22 High	6,603
SupT1 CD22 Mid	3,594
SupT1 CD22 Low	490

D

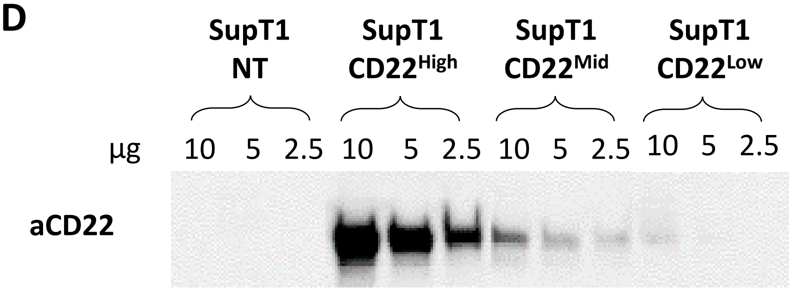

E

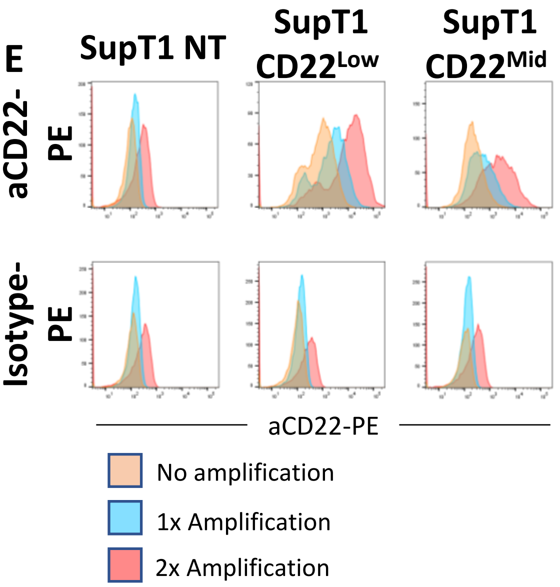

Figure S2: Engineered SupT1 cells to express high and low densities of CD22

A) In order to obtain low expressing CD22 target cells lines we introduced the STOP/SKIP technology, which comprises a STOP codon followed by a SKIP sequence leading to the ribosome skipping the STOP codon. The STOP/SKIP upstream of the transgene reduces the expression of the transgene. **B)** The expression of CD22 on the engineered SupT1 was measured by flow cytometry. The cells were labelled with either isotype control or aCD22 antibody, as well as the marker gene upstream of the transgene was labelled to ascertain the cassette expression. **C)** The antigen density was calculated based on the flow cytometry while concomitantly using with Quantibrite beads to obtain a standard curve. The CD22 level on SupT1 $CD22^{Low}$ was only subtly detectable by flow cytometry. **D)** Hence, we run western blot with different concentration of cell lysate loaded to investigate the presence of CD22 in SupT1 $CD22^{Low}$. **E)** Finally, in order to ascertain the presence of CD22 on the SupT1 by flow cytometry, we amplified the signal using QIFIKIT kit for one or two rounds of amplification.

Figure S3: Binder Screening

A

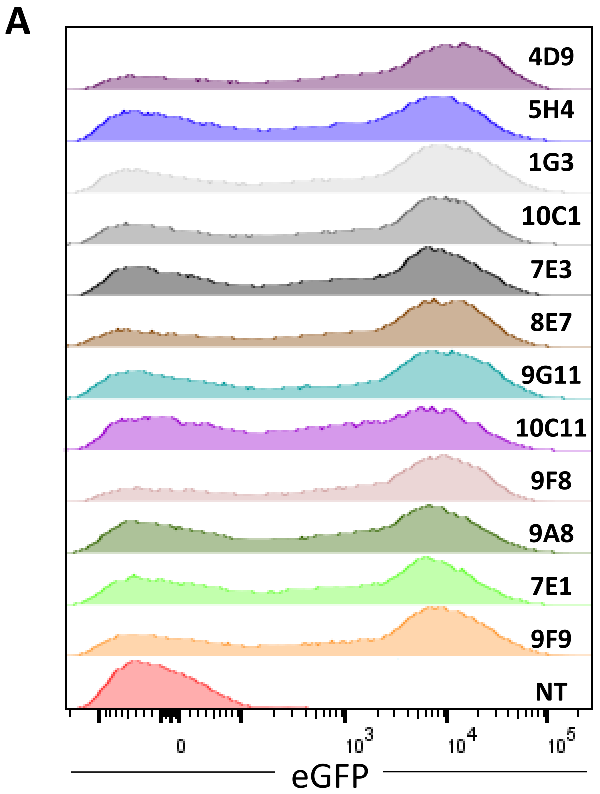

B

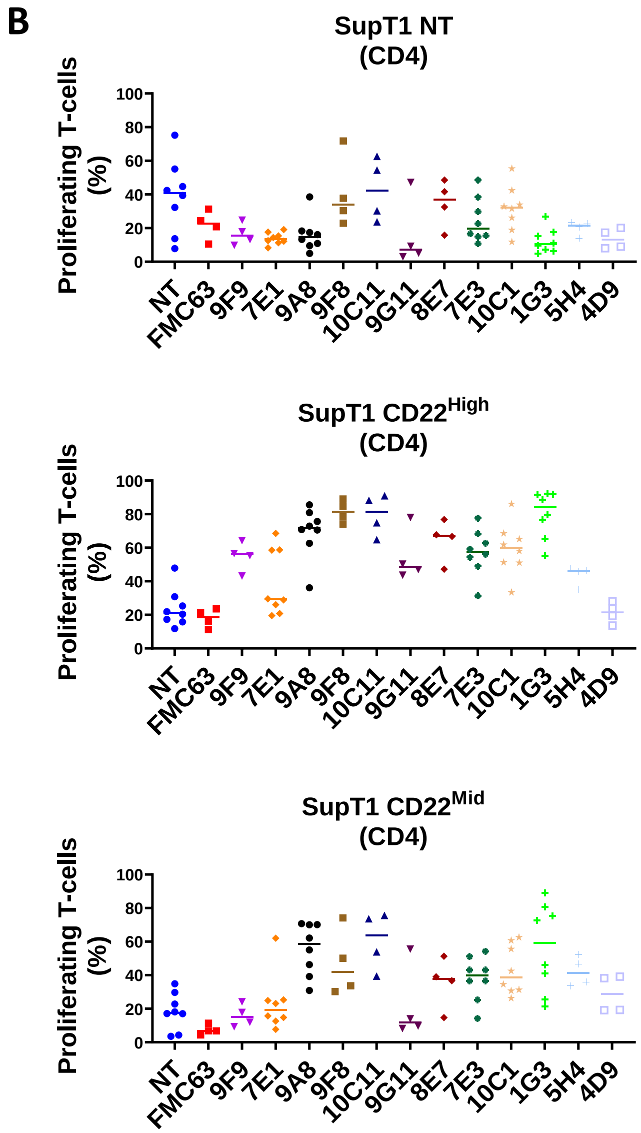

Figure S3: Binder Screening

A) The expression of the aCD22 binders was validated by the expression of the cassette marker gene, eGFP. **B)** T cells were labelled with CellTrace Violet and cultured in the presence of non-transduced SupT1 cells, or SupT1 engineered to express high and low levels of CD22 at a ratio of 1:1 for 4 days. The populations shown is the transduced proliferating CD4 compartment.

Figure S4: Comparison of 9A8 to M971 in lentiviral platforms

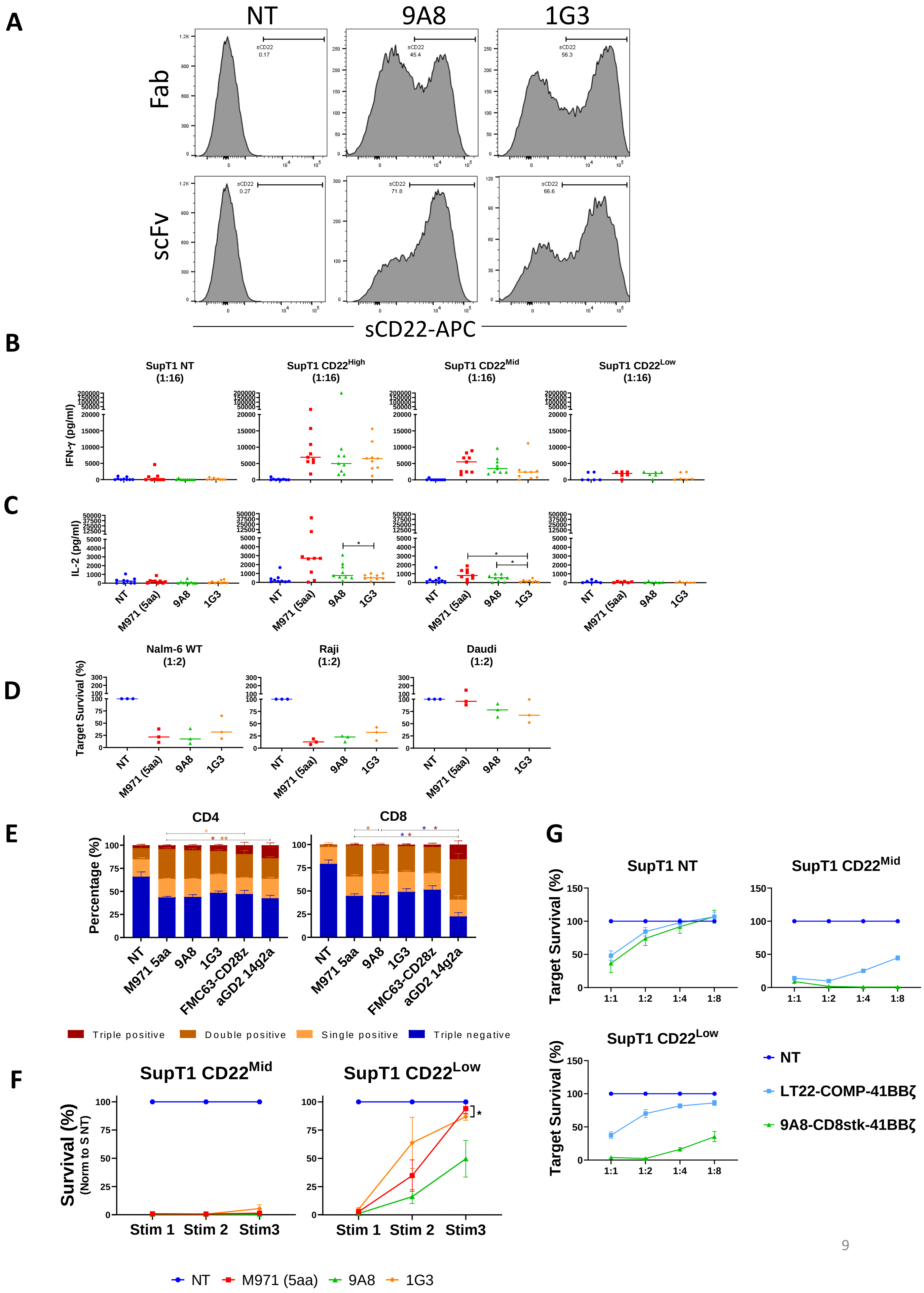

Figure S4: Comparison of 9A8 to M971 in lentiviral platforms

A) The expression of either scFv or Fab bearing CARs was assessed by the labelling with soluble CD22 antigen. The cytokine production of the M971, 9A8 and 1G3 CARs in the scFv lentiviral format was measured by ELISA for the ratio 1:16. The cytokines measured were IFN-γ **(B)** or IL-2 **(C)**. **D)** The CAR cytotoxicity was measured against B-cell lines, such as Nalm-6, Raji, and Daudi cells at an E:T of 1:2 at 72h. **E)** We assessed the exhaustion profile of the CARs by labelling for the exhaustion markers: PD-1, Tim3 and Lag3, and measuring the severity of exhaustion by the number of markers concomitantly expressed. The exhaustion read-out was flow-cytometry based on day 6 after a starvation assay. **F)** The CAR T cells were challenged in a re-stimulation assay, wherein the T cells were stimulated with SupT1 $CD22^{Mid}$ or $CD22^{Low}$ target cells three consecutive times. **G)** 9A8 was compared to LT22 at E:T ratios of 1:1, 1:2, 1:4, and 1:8. All statistical analyses were carried out with Student's t-tests. (n=3-9)

Figure S5: *In vivo* comparison of 9A8 to M971 in lentiviral platforms

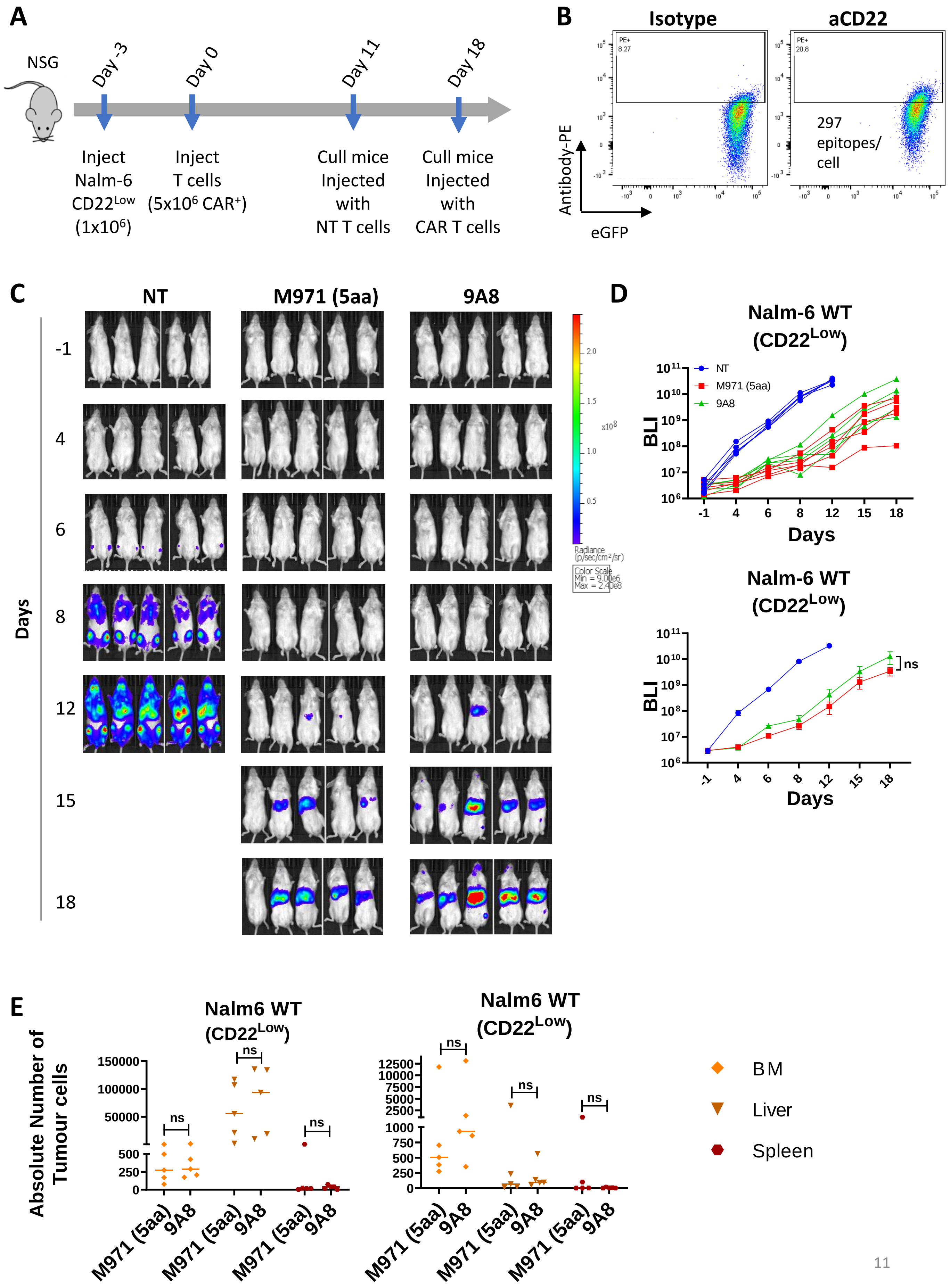

Figure S5: *In vivo* comparison of 9A8 to M971 in lentiviral platforms

A) Schematic representation of the *in vivo* NSG model. **B)** The CD22 antigen profile of Nalm-6 wt ($CD22^{Low}$) was validated by isotype or aCD22-PE labelling and measured with Quantibrite beads. BLI was performed to measure the tumour burden up to day 18 after T cells injection as shown in **(C)**. **D)** The BLI measurements were plotted to compare the anti-tumour efficacy between M971 and 9A8. **E)** The tumour cells and CAR T cells were enumerated by flow cytometry on day 18 for bone marrow, liver, and spleen tissues. All statistical analyses were carried out with Student's t-tests. (n=5 mice, T cells derived from 1 donor)

Figure S6: Co-transduction of CAT and 9A8

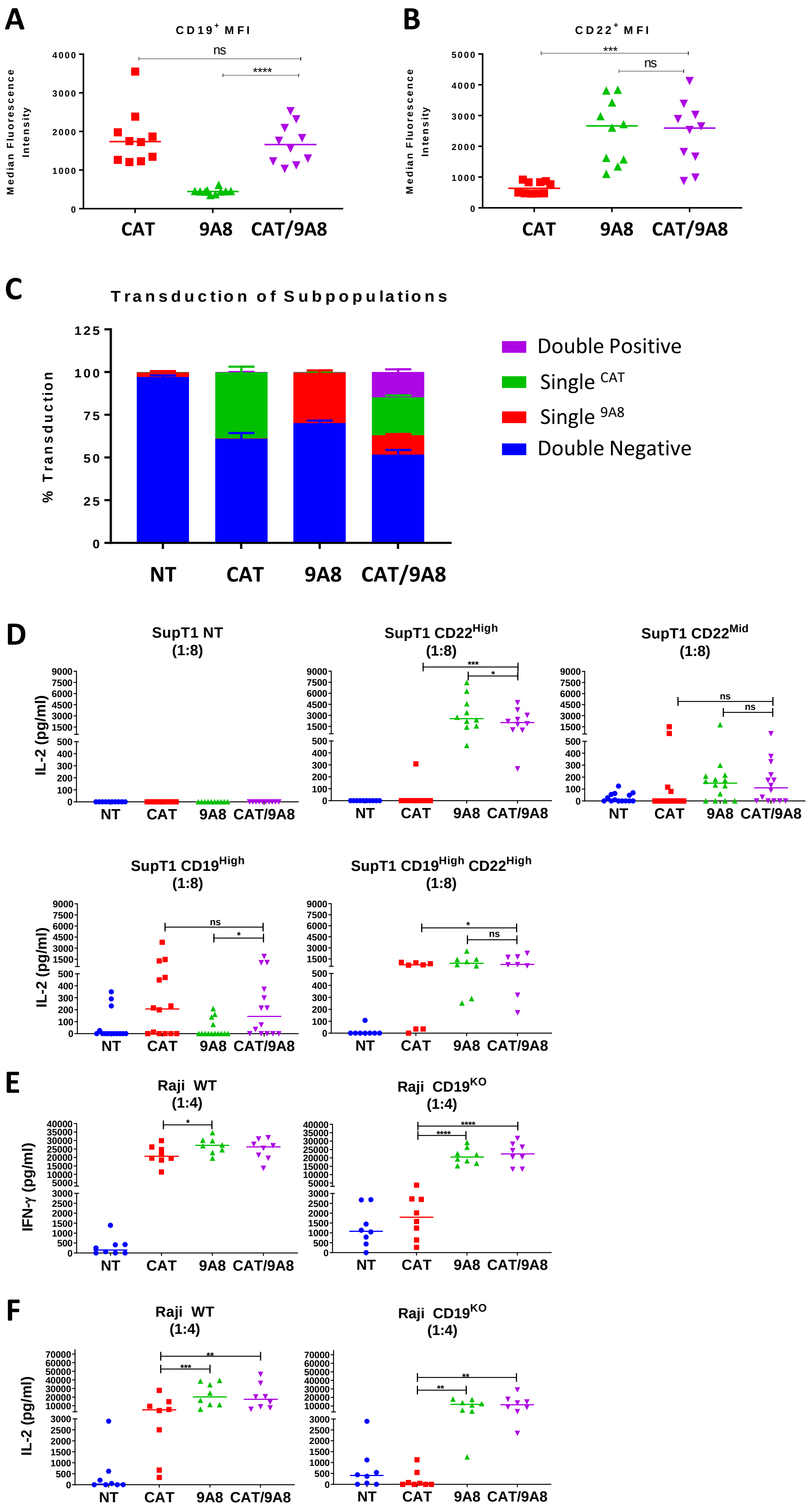

Figure S6: Co-transduction of CAT and 9A8

The MFI for the expression of the CAT **(A)** or 9A8 **(B)** CAR in the single or double transduction conditions was assessed by labelling the cells with anti-CAT idiotype and soluble CD22. The expression level of either CAR in the CAT/9A8 product was comparable to the single CAR conditions. The prevalence of each CAR subpopulation expressed in CAT/9A8 is shown in **(C)**, wherein Single^{CAT} or Single^{9A8} or double expressing subpopulations are shown in red, green or purple, respectively. **D)** The IL-2 release capacity was measured in response to CD22 mid or high expressing SupT1 targets at 1:8 E:T at a 72h time-point. Both IFN-γ **(E)** and IL-2 **(F)** secretion of the CARs against Raji WT and Raji CD19^{KO} (1:2 E:T) cells was quantified by ELISA at 72h. All statistical analyses were carried out with Student's t-tests. (n=8)

Figure S7: Engineering Nalm-6 in order to knock-out CD19

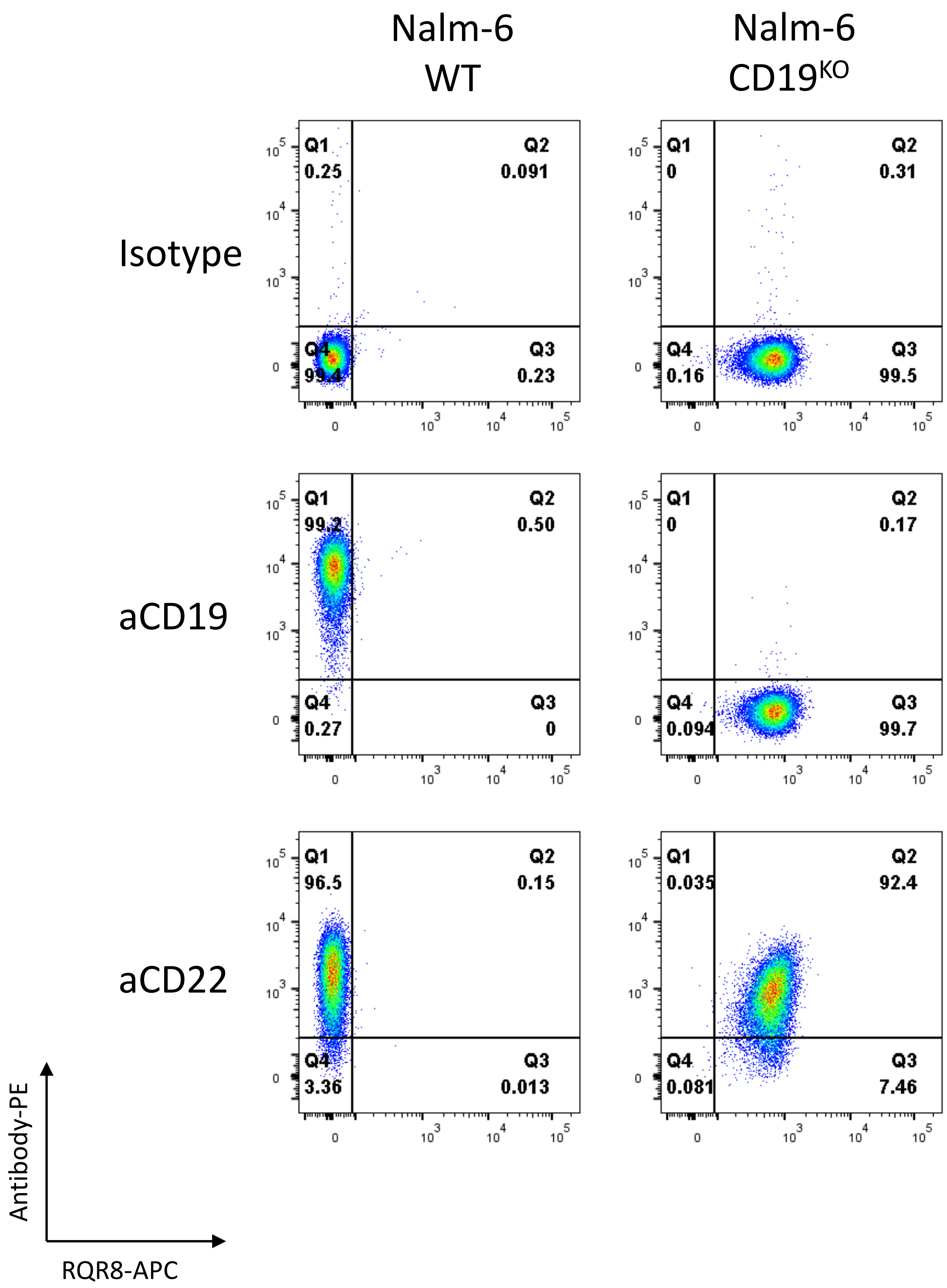

Figure S7: Engineering Nalm-6 in order to knock-out CD19

CD19 was downregulated in Nalm-6 WT cells by CRISPR technology. Flow-cytometry assessment demonstrated the lack of CD19 expression in the engineered Nalm-6 $CD19^{KO}$ cells, while the expression of CD22 antigen is still present.

Figure S8: *In vivo* data for CAT/9A8

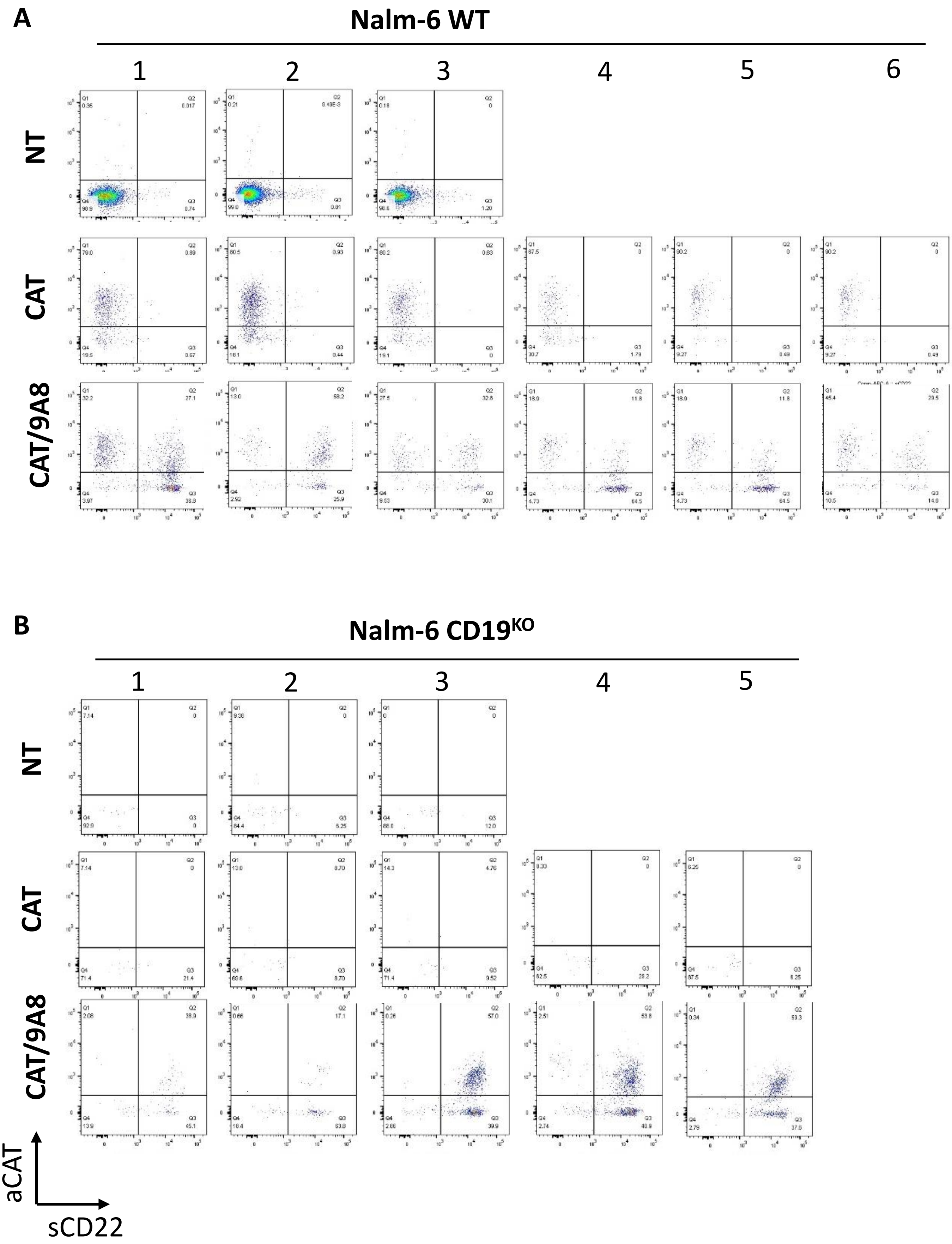

Figure S8: *In vivo* data for CAT/9A8

A) NSG mice were injected with 10^6 Nalm-6 WT cells at day -4 and at day 0 were inoculated with $5x10^6$ transduced CAR T cells. **B)** Nalm-6 $CD19^{KO}$ were utilised to simulate an antigen escape model. 10^6 tumour cells were injected 8 days prior to the T cell inoculation of $5x10^6$ transduced CAR T cells. Bone marrow from mice culled at day 14 were homogenised and labelled for the CAR bearing human T cells. The CAR expression was validated by labelling with anti-CAT idiotype and soluble CD22 for both Nalm-6 WT **(A)** and $CD19^{KO}$ **(B)** cells.
